# Association of Adverse Effects of Medical Treatment With Mortality in the United States: A Secondary Analysis of the Global Burden of Diseases, Injuries, and Risk Factors Study

**DOI:** 10.1001/jamanetworkopen.2018.7041

**Published:** 2019-01-18

**Authors:** Jacob E. Sunshine, Nicholas Meo, Nicholas J. Kassebaum, Michael L. Collison, Ali H. Mokdad, Mohsen Naghavi

**Affiliations:** 1 Department of Anesthesiology & Pain Medicine, University of Washington School of Medicine, Seattle; 2 Institute for Health Metrics and Evaluation, University of Washington, Seattle; 3 Pediatric Anesthesiology and Pain Medicine, Seattle Children’s Hospital, University of Washington School of Medicine, Seattle; 4 Department of Medicine, University of Washington School of Medicine, Seattle

## Abstract

**Importance:**

More than 20 years have passed since the first publication of estimates of the extent of medical harm occurring in hospitals in the United States. Since then, considerable resources have been allocated to improve patient safety, yet policymakers lack a clear gauge of the progress made.

**Objectives:**

To quantify the cause-specific mortality associated with adverse effects of medical treatment (AEMT) in the United States from 1990 to 2016 by age group, sex, and state of residence and to describe trends in types of harm and associations with other diseases and injuries.

**Design, Setting, and Participants:**

Cohort study using 1990-2016 data on mortality due to AEMT from the Global Burden of Diseases, Injuries, and Risk Factors (GBD) 2016 study, which assessed death certificates of US decedents.

**Exposures:**

Death with *International Classification of Diseases* (*ICD*)–coded registration.

**Main Outcomes and Measures:**

Mortality associated with AEMT. Secondary analyses were performed on all *ICD* codes in the death certificate’s causal chain to describe associations between AEMT and other diseases and injuries.

**Results:**

From 1990 to 2016, there were an estimated 123 603 deaths (95% uncertainty interval [UI], 100 856-163 814 deaths) with AEMT as the underlying cause. Despite an overall increase in the number of deaths due to AEMT over time, the national age-standardized mortality rate due to AEMT decreased by 21.4% (95% UI, 1.3%-32.2%) from 1.46 (95% UI, 1.09-1.76) deaths per 100 000 population in 1990 to 1.15 (95% UI, 1.00-1.60) deaths per 100 000 population in 2016. Men and women had similar rates of AEMT mortality, and those 70 years or older had mortality rates nearly 20-fold greater compared with those aged 15 to 49 years (mortality rate in 2016 for both sexes, 7.93 [95% UI, 7.23-11.45] per 100 000 population for those aged ≥70 years vs 0.38 [95% UI, 0.34-0.43] per 100 000 population for those aged 15-49 years). Per 100 000 population, California had the lowest age-standardized AEMT mortality rate at 0.84 deaths (95% UI, 0.57-1.47 deaths), whereas Mississippi had the highest mortality rate at 1.67 deaths (95% UI, 1.19-2.03 deaths). Surgical and perioperative events were the most common subtype of AEMT, accounting for 63.6% of all deaths for which an AEMT was identified as the underlying cause.

**Conclusions and Relevance:**

This study’s findings suggest a modest reduction in the mortality rate associated with AEMT in the United States from 1990 to 2016 while also observing increased mortality associated with advancing age and noted geographic variability. The annual GBD releases may allow for tracking of the burden of AEMT in the United States.

## Key Points

**Question:**

How has mortality associated with adverse effects of medical treatment in the United States changed over time, by state, age, and sex?

**Findings:**

In this cohort study, there was a decrease in the national age-standardized mortality rate associated with adverse effects of medical treatment in the United States between 1990 and 2016. Although no differences by sex were observed, increased mortality due to adverse effects of medical treatment was associated with advancing age, and geographic variability was noted.

**Meaning:**

Global Burden of Disease 2016 results suggest that mortality associated with the adverse effects of medical treatment has decreased modestly over the past 25 years, and although the degree of improvement varies by state, it appears that an increased burden of adverse effects of medical treatment on aging populations continues to affect the US health system.

## Teaser

This cohort study quantifies the cause-specific mortality associated with adverse effects of medical treatment in the United States from 1990 to 2016 and describes trends in types of harm and associations with other diseases and injuries.

## Introduction

More than 20 years ago, the Harvard Medical Practice Study provided the first estimate of the extent of medical harm occurring in US hospitals.^1^ Building on mounting evidence from several studies, the Institute of Medicine (IOM) published their landmark report, *To Err Is Human: Building a Safer Health System*, in 1999, in which they estimated that approximately 44 000 to 98 000 deaths occur annually because of medical errors.^2^

Considerable resources have been allocated to improve patient safety since these reports on patient harm, with significant advances achieved in safety research, quality improvement initiatives, policy, health information technology, reimbursement strategies, and accreditation standards.^2,3,4,5,6,7,8,9,10^ In certain clinical domains, such as hospital-acquired infections and transitions of care between teams and patient care units, there have been several reports suggesting notable nationwide improvements in outcomes.^11,12,13^ It is unclear, however, if improvements in such proxy measures of overall patient safety have translated into mortality improvements across all types of adverse effects of medical treatment (AEMT) over time.

Although adverse event detection methods have continued to advance in recent years, a significant challenge remains in gauging the progress made at the state or national level. Previous studies of medical adverse events have been crucial to both jumpstarting the patient safety movement and to providing critical insights into medical harm, but commonly applied approaches are limited in several key respects. First, point estimates of medical harm using retrospective surveillance systems—several of which have reported higher estimates of annual mortality related to medical errors than the IOM report—are derived from resource-intensive medical record reviews.^1,14,15,16,17,18,19,20,21,22^ These tools are excellent for use at a health care organizational level but make comprehensive and consistently applied assessments on a state or national scale particularly challenging. Second, voluntary reporting systems may be used to monitor patient safety trends on a larger scale but are known to have selection bias and underreporting.^23,24,25,26^ Finally, administrative databases screened for adverse events may be limited to a range of conditions or have an overall low detection sensitivity.^27,28,29,30^

The Global Burden of Diseases, Injuries, and Risk Factors (GBD) study^31^ is the most comprehensive source of comparable information on the levels and trends of health loss across all disease and injuries throughout the world. This report uses the 2016 GBD study to present the scope and trend of mortality associated with AEMT in the United States from 1990 to 2016.

## Methods

### 2016 GBD Study

The 2016 GBD study is a multinational collaborative project with an aim of providing regular and consistent estimates of health loss worldwide. Methods for GBD 2016 have been reported in full elsewhere.^31^ Briefly, data were obtained from deidentified death records from the National Center for Health Statistics^32^; records included information on sex, age, state of residence at time of death, and underlying cause of death. Causes were classified according to the *International Classification of Diseases, Ninth Revision* (*ICD-9*), for deaths prior to 1999 and the *International Statistical Classification of Diseases and Related Health Problems, Tenth Revision* (*ICD-10*) for subsequent deaths.^33,34^ Each death was categorized as resulting from a single underlying cause. All *ICD* codes were mapped to the GBD cause list, which is hierarchically organized, mutually exclusive, and collectively exhaustive.^31^ The complete lists of *ICD-9* and *ICD-10* codes mapped to AEMT in the 2016 GBD study cause classification system are in eTables 1 and 2 in the Supplement. This cohort study followed the Strengthening the Reporting of Observational Studies in Epidemiology (STROBE) reporting guidelines. This research received institutional review board approval from the University of Washington, Seattle. Because anonymized death records were analyzed retrospectively, informed consent was not required. We defined AEMT as a coded injury that arises from an individual’s medical management.

### Mortality Rates

Cause-of-death ensemble modeling (CODEm), a standard analytic tool used in GBD cause-specific mortality analyses, was used to generate mortality rate and cause fraction (percentage of all-cause deaths due to a specific GBD cause) estimates for the years 1990 through 2016. A full technical description of CODEm based on the most up-to-date iteration of the GBD methodology^31,35^ is available in the eMethods and eTables 3 and 4 in the Supplement. Results are age standardized using the GBD world age population standard, which accounts for differences in population size and age structure.^31,36^

The GBD methodology also accounts for when ill-defined or implausible causes were coded as the underlying cause of death.^37^ Plausible underlying causes of death were assigned to each ill-defined or implausible cause of death according to proportions derived in 1 of 3 ways: (1) published literature or expert opinion, (2) regression models, and (3) initial proportions observed among targets. These codes are shown in eTable 4 in the Supplement.

### Statistical Analysis

All estimates in the primary analysis include an uncertainty interval (UI), which represents a range of values that reflects the certainty of an estimate. The UIs are generated by sampling 1000 values (called *draws*) for each estimate and summing the draws across age, cause, and location for all intermediate calculations. The UIs are defined by the 25th and 975th draw values, representing the 2.5th and 97.5th percentiles.

### AEMT Subtypes

Two secondary analyses were completed as part of this report to supplement the GBD 2016 findings. First, the *ICD* codes encompassing AEMT were subdivided into categories to capture the nature of the medical harm based on aggregate analysis of all available death records from 1980 to 2014 (N = 80 414 952). The *ICD* classification framework adopted for this secondary analysis was modified from Houghland et al^38^ and allocated AEMT deaths into 6 categories: (1) adverse drug events, (2) surgical and perioperative adverse events, (3) misadventure (events likely to represent medical error, such as accidental laceration or incorrect dosage), (4) adverse events associated with medical management, (5) adverse events associated with medical or surgical devices, and (6) other. The categorization of *ICD* codes into the 6 subgroups was done by consensus among 3 of us (J.E.S., N.M., and M.N.). All subcategorized codes are available in eTables 1 and 2 in the Supplement.

### Cause-of-Death Chain Analysis

The final secondary analysis involved the analysis of the cause-of-death chains for all deaths from 1980 to 2014 to measure how frequently AEMT was (1) anywhere within a death certificate’s cause-of-death chain (ie, not underlying cause) and (2) which other contributing causes were most frequently found in the causal chain when AEMT was certified as the underlying cause. Because multiple causes can appear in the causal chain for any single death, the results of this analysis were not mutually exclusive. The secondary analysis involves actual counts and does not represent a modeled estimate; as such, the results are from 1980 through 2014 (the earliest to the most recent year in which data were available for this analysis).

## Results

**Figure 1.**
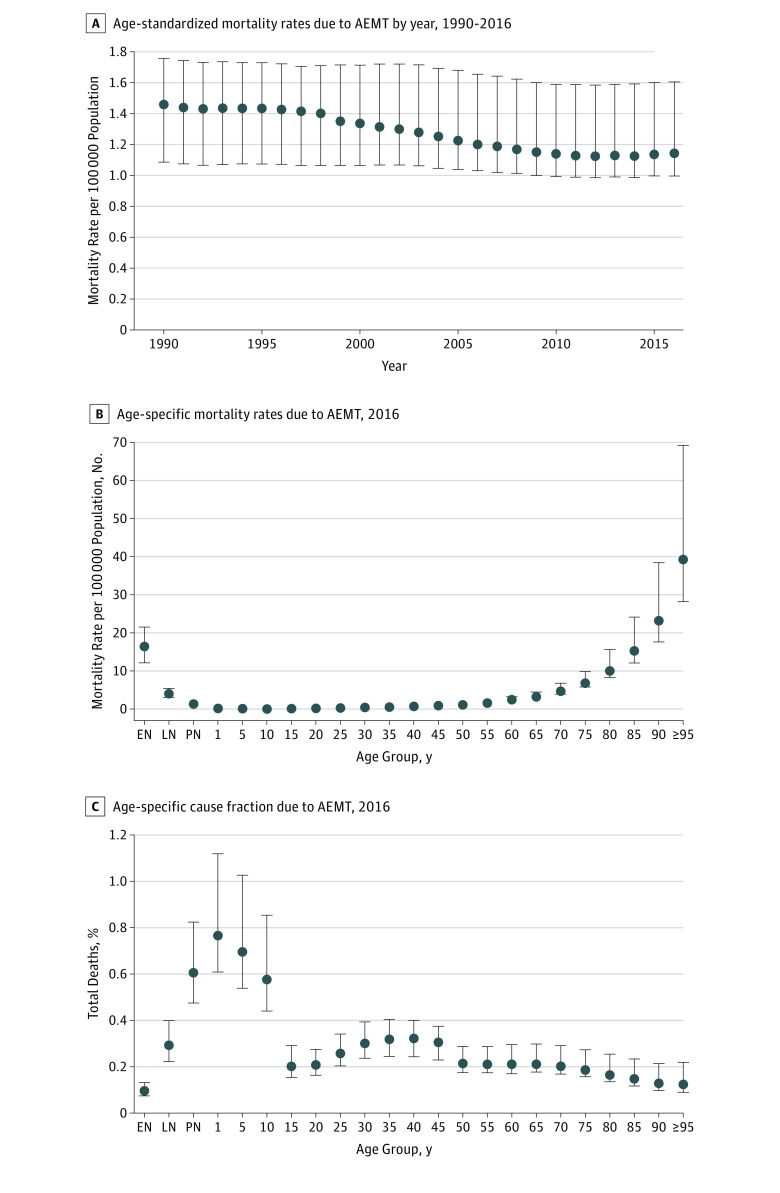
Trend in Mortality Rate and Cause Fraction Associated With Adverse Effects of Medical Treatment (AEMT), United States Results are for both sexes combined. Data source: the 2016 Global Burden of Diseases, Injuries, and Risk Factors study.^31^ Error bars indicate 95% uncertainty intervals. Cause fraction indicates the percentage of all-cause deaths due to a specific global burden of disease cause. EN indicates early neonatal period (0-6 days); LN, late neonatal period (7-28 days); and PN, postneonatal period (1-12 months).

From 1990 to 2016 in the United States, there were 123 603 deaths (95% UI, 100 856-163 814 deaths) in which AEMT represented the underlying cause of death. The absolute number of deaths in which AEMT was the underlying cause increased from 4180 (95% UI, 3087-4993) in 1990 to 5180 (95% UI, 4469-7436) in 2016. Most of this increase was due to population growth and aging, as demonstrated by a 21.4% decrease (95% UI, 1.3%-32.2%) in the national age-standardized AEMT mortality rate over the same period, from 1.46 (95% UI, 1.09-1.76) deaths per 100 000 population in 1990 to 1.15 (95% UI, 1.00-1.60) deaths per 100 000 population in 2016 (Figure 1A). When not exclusively measured as the underlying cause of death, AEMT appeared in the cause-of-death chain in 2.7% of all deaths from 1980 to 2014, which corresponds to AEMT being a contributing cause for an additional 20 deaths for each death when it is the underlying cause. Mortality associated with AEMT as either an underlying or contributing cause appeared in 2.8% of all deaths.

The AEMT mortality rates among men and women were similar (not shown), but there were considerable differences in AEMT mortality with respect to age at death. Per 100 000 population, older individuals had higher mortality rates associated with AEMT compared with younger populations: aged 15-49 years, 0.38 (95% UI, 0.34-0.43) deaths; aged 50-69 years, 1.68 (95% UI, 1.57-2.07) deaths; 70 years or older, 7.93 (95% UI, 7.23-11.45) deaths (Figure 1B). However, when looking at the AEMT mortality cause fraction, children, adolescents, and young adults had a proportionally higher mortality burden from AEMT than older adults (Figure 1C).

**Figure 2.**
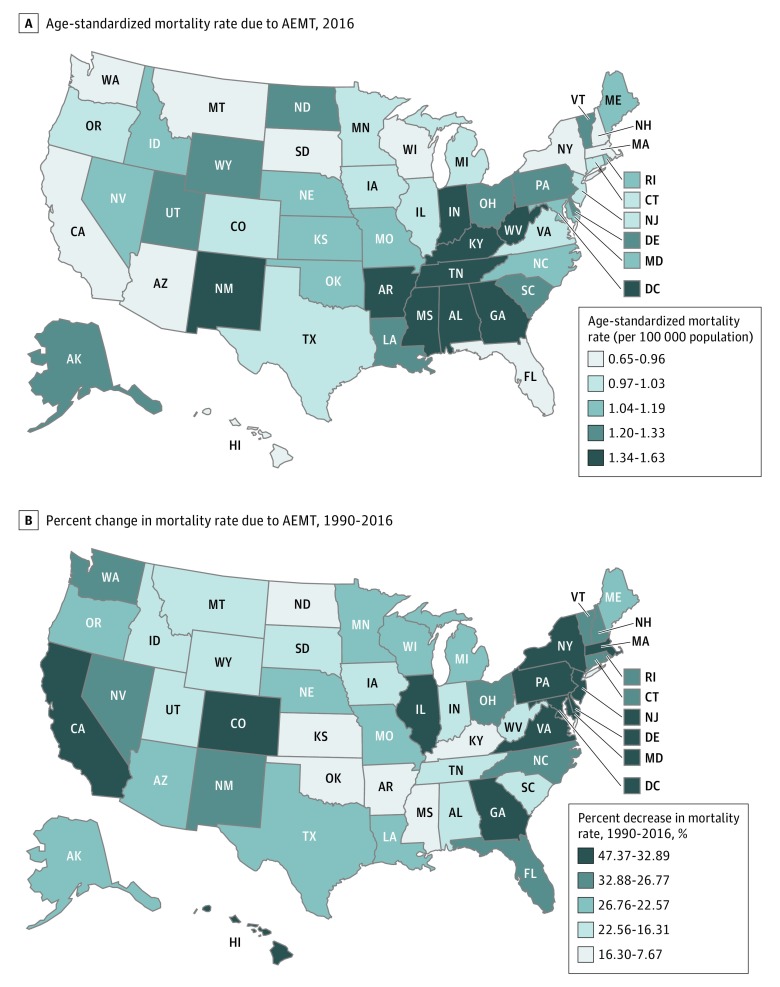
State-Level Mortality Associated With Adverse Effects of Medical Treatment (AEMT), United States Results are for both sexes combined. Data source: the 2016 Global Burden of Diseases, Injuries, and Risk Factors study.^31^

Interstate variability in AEMT mortality was observed (Table 1 and Figure 2A). In 2016, California had the lowest age-standardized AEMT mortality rate at 0.84 (95% UI, 0.57-1.47) deaths per 100 000 population, whereas Mississippi had the highest at 1.67 (95% UI, 1.19-2.03) deaths per 100 000 population (Table 1). From 1990 to 2016, mortality estimates in many states decreased. The largest percentage decrease in age-standardized AEMT mortality rate occurred in the District of Columbia (39.9%) followed by New York (33.0%), Maryland (32.2%), New Jersey (30.5%), and California (28.5%) (Table 1 and Figure 2B). In addition, Colorado (16.6%), Oregon (14%), and Virginia (18.8%) all had decreased AEMT mortality after the year 2000.

**Table 1.** Age-Standardized Mortality Rate Due to Adverse Effects of Medical Treatment in 1990, 2000, and 2016 and the Percentage Change Over Time, Nationally and by State in the United States

Location	Age-Standardized Mortality Rate per 100 000 Population (95% UI)			Percentage Change, % (95% UI)	
	1990	2000	2016	1990-2016	2000-2016
United States	1.46 (1.09 to 1.76)	1.34 (1.06 to 1.71)	1.15 (1.00 to 1.60)	−21.4 (−32.2 to −1.3)	−14.3 (−22.4 to −1.8)
Alabama	1.78 (1.17 to 2.09)	1.69 (1.20 to 1.94)	1.55 (1.20 to 1.98)	−12.8 (−28.9 to 11.7)	−8.0 (−22.8 to 9.8)
Alaska	1.65 (1.18 to 1.88)	1.49 (1.13 to 1.81)	1.33 (1.08 to 1.79)	−19.5 (−36.1 to 3.9)	−11.3 (−26.9 to 6.2)
Arizona	1.37 (1.06 to 1.71)	1.28 (1.04 to 1.70)	1.10 (0.89 to 1.58)	−20.3 (−35.2 to 1.3)	−14.7 (−27.6 to 0.2)
Arkansas	1.58 (1.15 to 1.89)	1.56 (1.17 to 1.88)	1.45 (1.16 to 1.93)	−8.0 (−22.4 to 9.9)	−7.2 (−20.6 to 7.0)
California	1.18 (0.97 to 1.70)	0.99 (0.73 to 1.61)	0.84 (0.57 to 1.47)	−28.5 (−45.6 to −5.6)	−15.1 (−28.1 to −1.3)
Colorado	1.43 (1.01 to 1.65)	1.33 (1.02 to 1.63)	1.11 (0.91 to 1.53)	−22.5 (−37.4 to 2.1)	−16.6 (−29.5 to −1.0)
Connecticut	1.46 (1.01 to 1.67)	1.36 (0.98 to 1.58)	1.07 (0.86 to 1.44)	−26.8 (−41.9 to 1.0)	−21.3 (−36.3 to 1.8)
Delaware	1.74 (1.14 to 2.03)	1.56 (1.10 to 1.78)	1.29 (0.99 to 1.63)	−26.1 (−39.6 to −1.9)	−17.5 (−29.6 to −0.4)
District of Columbia	2.47 (1.29 to 3.11)	2.05 (1.18 to 2.53)	1.48 (0.98 to 1.79)	−39.9 (−51.6 to −11.5)	−27.6 (−40.4 to −3.0)
Florida	1.17 (0.99 to 1.65)	1.07 (0.85 to 1.63)	0.95 (0.70 to 1.55)	−18.3 (−34.4 to 0.9)	−10.7 (−22.5 to 0.3)
Georgia	1.91 (1.17 to 2.31)	1.86 (1.17 to 2.21)	1.44 (1.06 to 1.74)	−24.5 (−40.0 to 4.8)	−22.4 (−37.6 to 1.8)
Hawaii	1.07 (0.90 to 1.49)	0.98 (0.81 to 1.43)	0.86 (0.66 to 1.37)	−19.7 (−34.6 to −3.3)	−11.9 (−24.7 to 1.0)
Idaho	1.46 (1.02 to 1.68)	1.38 (1.03 to 1.66)	1.24 (1.00 to 1.66)	−15.4 (−32.0 to 9.5)	−10.6 (−25.6 to 6.9)
Illinois	1.53 (1.11 to 1.80)	1.38 (1.08 to 1.74)	1.12 (0.92 to 1.60)	−26.6 (−41.5 to −2.1)	−18.5 (−31.8 to −1.1)
Indiana	1.63 (1.11 to 1.88)	1.66 (1.13 to 1.92)	1.43 (1.12 to 1.78)	−12.3 (−27.8 to 9.7)	−14.1 (−29.5 to 6.0)
Iowa	1.24 (1.00 to 1.60)	1.16 (0.98 to 1.58)	1.09 (0.88 to 1.62)	−11.4 (−27.0 to 8.9)	−6.0 (−19.0 to 7.4)
Kansas	1.36 (1.03 to 1.66)	1.35 (1.06 to 1.70)	1.23 (0.99 to 1.69)	−9.7 (−25.9 to 10.9)	−9.2 (−23.8 to 6.9)
Kentucky	1.61 (1.16 to 1.91)	1.58 (1.19 to 1.94)	1.47 (1.18 to 1.92)	−8.5 (−22.6 to 9.5)	−6.4 (−17.6 to 6.3)
Louisiana	1.74 (1.21 to 1.99)	1.63 (1.21 to 1.99)	1.46 (1.19 to 1.95)	−15.7 (−30.5 to 6.2)	−10.3 (−22.0 to 4.3)
Maine	1.47 (1.07 to 1.76)	1.37 (1.08 to 1.72)	1.19 (1.00 to 1.62)	−18.9 (−33.3 to 2.2)	−12.9 (−25.4 to 0.6)
Maryland	1.81 (1.14 to 2.16)	1.58 (1.09 to 1.80)	1.23 (0.96 to 1.57)	−32.2 (−46.0 to −3.4)	−22.2 (−34.3 to −2.3)
Massachusetts	1.31 (1.04 to 1.68)	1.14 (0.95 to 1.64)	0.99 (0.76 to 1.50)	−24.3 (−40.7 to 0.1)	−12.9 (−25.1 to 0.5)
Michigan	1.40 (1.12 to 1.80)	1.32 (1.10 to 1.77)	1.18 (0.97 to 1.68)	−15.7 (−29.3 to 1.0)	−10.6 (−22.1 to 0.8)
Minnesota	1.37 (0.98 to 1.59)	1.27 (0.99 to 1.57)	1.10 (0.90 to 1.55)	−19.6 (−35.4 to 6.1)	−13.3 (−26.8 to 2.9)
Mississippi	1.76 (1.20 to 2.04)	1.81 (1.22 to 2.09)	1.67 (1.19 to 2.03)	−5.3 (−20.2 to 10.1)	−7.9 (−22.0 to 8.6)
Missouri	1.44 (1.09 to 1.78)	1.40 (1.13 to 1.82)	1.23 (1.03 to 1.75)	−14.7 (−30.0 to 5.9)	−11.8 (−24.3 to 1.4)
Montana	1.27 (1.04 to 1.67)	1.21 (1.02 to 1.69)	1.07 (0.83 to 1.60)	−15.9 (−33.0 to 7.0)	−12.0 (−27.4 to 6.9)
Nebraska	1.41 (1.03 to 1.64)	1.34 (1.03 to 1.65)	1.19 (0.99 to 1.64)	−15.3 (−30.7 to 9.1)	−10.9 (−23.8 to 5.2)
Nevada	1.59 (1.18 to 1.91)	1.45 (1.15 to 1.85)	1.22 (1.00 to 1.70)	−22.8 (−37.5 to −0.3)	−15.8 (−28.7 to −1.0)
New Hampshire	1.34 (1.05 to 1.71)	1.20 (1.01 to 1.63)	1.05 (0.84 to 1.55)	−21.9 (−36.8 to 0.3)	−12.7 (−25.7 to 1.9)
New Jersey	1.58 (1.10 to 1.80)	1.34 (1.03 to 1.69)	1.10 (0.89 to 1.50)	−30.5 (−45.1 to −4.0)	−18.4 (−30.9 to −2.1)
New Mexico	1.98 (1.07 to 2.55)	1.82 (1.03 to 2.28)	1.64 (1.01 to 2.05)	−17.3 (−31.7 to 5.1)	−9.9 (−23.5 to 8.1)
New York	1.42 (1.10 to 1.78)	1.16 (0.99 to 1.60)	0.95 (0.74 to 1.45)	−33.0 (−47.8 to −8.2)	−17.9 (−31.8 to −1.7)
North Carolina	1.65 (1.12 to 1.91)	1.53 (1.13 to 1.83)	1.30 (1.07 to 1.70)	−21.1 (−36.1 to 4.7)	−15.0 (−27.6 to 0.7)
North Dakota	1.38 (0.95 to 1.59)	1.29 (0.96 to 1.54)	1.16 (0.92 to 1.53)	−15.8 (−31.6 to 10.2)	−10.2 (−23.7 to 8.7)
Ohio	1.62 (1.10 to 1.85)	1.50 (1.12 to 1.81)	1.31 (1.08 to 1.74)	−19.0 (−33.9 to 4.5)	−12.3 (−24.5 to 2.0)
Oklahoma	1.41 (1.14 to 1.83)	1.44 (1.18 to 1.91)	1.35 (1.13 to 1.92)	−4.4 (−18.1 to 11.6)	−6.4 (−18.5 to 6.3)
Oregon	1.35 (1.07 to 1.73)	1.27 (1.07 to 1.73)	1.10 (0.88 to 1.62)	−18.6 (−33.7 to 2.4)	−14.0 (−26.2 to −0.1)
Pennsylvania	1.76 (1.09 to 2.09)	1.58 (1.08 to 1.82)	1.28 (1.01 to 1.62)	−27.1 (−41.8 to 0.7)	−19.2 (−32.5 to 0.7)
Rhode Island	1.48 (1.05 to 1.71)	1.33 (1.02 to 1.63)	1.15 (0.93 to 1.57)	−22.1 (−37.8 to 1.1)	−13.2 (−27.3 to 2.7)
South Carolina	1.57 (1.15 to 1.92)	1.54 (1.17 to 1.88)	1.37 (1.11 to 1.83)	−13.3 (−27.8 to 7.8)	−11.6 (−26.3 to 6.7)
South Dakota	1.18 (1.00 to 1.59)	1.16 (0.97 to 1.63)	1.05 (0.80 to 1.61)	−11.5 (−29.2 to 13.1)	−9.4 (−23.9 to 7.4)
Tennessee	1.62 (1.16 to 1.88)	1.62 (1.19 to 1.94)	1.47 (1.16 to 1.88)	−9.6 (−23.7 to 9.7)	−9.2 (−21.4 to 4.5)
Texas	1.38 (1.10 to 1.77)	1.31 (1.09 to 1.74)	1.17 (0.96 to 1.65)	−15.3 (−28.6 to 4.4)	−11.1 (−22.1 to 1.7)
Utah	1.56 (0.99 to 1.85)	1.51 (1.03 to 1.74)	1.35 (0.99 to 1.65)	−13.7 (−29.4 to 10.8)	−10.8 (−23.5 to 4.4)
Vermont	1.59 (1.05 to 1.85)	1.40 (1.00 to 1.62)	1.21 (0.96 to 1.55)	−23.6 (−38.5 to 1.4)	−13.8 (−26.9 to 2.3)
Virginia	1.56 (1.11 to 1.82)	1.44 (1.08 to 1.73)	1.17 (0.97 to 1.62)	−25.1 (−40.5 to 0.7)	−18.8 (−32.0 to −0.2)
Washington	1.17 (0.98 to 1.70)	1.06 (0.82 to 1.67)	0.94 (0.68 to 1.58)	−19.7 (−36.4 to 1.8)	−11.2 (−22.9 to 0.1)
West Virginia	1.63 (1.16 to 1.90)	1.58 (1.19 to 1.94)	1.51 (1.22 to 1.99)	−7.4 (−22.2 to 13.1)	−4.6 (−17.0 to 9.0)
Wisconsin	1.18 (1.00 to 1.67)	1.12 (0.92 to 1.65)	0.98 (0.71 to 1.59)	−17.2 (−33.7 to 4.5)	−13.2 (−28.1 to 2.0)
Wyoming	1.65 (1.06 to 1.95)	1.57 (1.07 to 1.81)	1.36 (1.01 to 1.66)	−17.3 (−32.7 to 7.5)	−13.3 (−27.6 to 5.7)

Abbreviation: UI, uncertainty interval.

**Figure 3.**
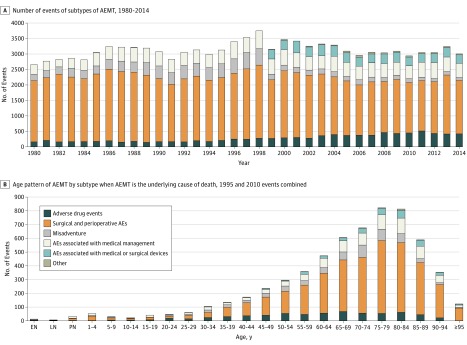
Subtypes of Adverse Effects of Medical Treatment (AEMT) Mortality A, Data include all ages and both sexes. B, Data include both sexes in 1995 and 2010. Misadventures are events likely to represent medical error, such as an accidental laceration or incorrect dosage. AEs indicates adverse events.

In the secondary analysis, in which AEMT was listed as the underlying cause of death, 8.9% were due to adverse drug events, 63.6% to surgical and perioperative adverse events, 8.5% to misadventure, 14% to adverse events associated with medical management, 4.5% to adverse events associated with medical or surgical devices, and 0.5% to other AEMT (eTable 6 in the Supplement). The ranking of the subtypes was stable over time (Figure 3A) but with increasing rates of adverse drug events and decreasing rates of misadventure and surgical and perioperative adverse events. Adverse events related to medical or surgical devices and other AEMT were nearly absent in the 1990s but have been responsible for a stable proportion of overall AEMT since the switch to *ICD-10* coding of death certificates. Surgical and perioperative adverse events were the most common subtype of AEMT in almost all age groups and increased in importance with age (Figure 3B); misadventure was the largest subtype in neonates, and adverse drug events predominated in individuals aged 20 to 24 years. For full results for when AEMT was the underlying cause of death, see eTables 5 and 6 in the Supplement.

In the final secondary analysis, AEMT as a contributing cause (ie, not underlying cause) of death was examined. Among the 2.7% of all deaths in which AEMT was a contributing cause, surgical and perioperative adverse effects were the most common subtype of AEMT, followed by misadventure (Table 2). Adverse drug events were the biggest contributor in children, adolescents, and elderly individuals, whereas surgical and perioperative adverse events were the most important in neonates and infants and were of increasing importance in older adults. When AEMT was not listed as the underlying cause of death, external injuries were the most commonly associated with AEMT, appearing in the cause-of-death chain of more than 20% of such deaths (eTable 7 in the Supplement).

**Table 2.** Rate of Occurrence of Other Nonunderlying Causes of Death Appearing in the Cause-of-Death Chain by Adverse Effects of Medical Treatment (AEMT) Category When AEMT Was Certified as the Underlying Cause of Death, 1980 to 2014, United States^a^

Nonunderlying Causes of Death	Occurrence Rate of Adverse Effects of Medical Treatment by Type per 1000 AEMT Deaths						All Types of AEMT per 1000 Instances of AEMT as Underlying Cause	Death Totals of Other Causes in the Chain When AEMT Is the Underlying Cause of Death
	Adverse Drug Events	Surgical and Perioperative Adverse Events	Misadventure^b^	Adverse Events Due to Medical Management	Adverse Events Due to Medical or Surgical Devices	Other		
External cause of injuries	464.74	307.25	325.17	391.34	960.72	127.21	363.02	39 620
Chronic infectious disease	7.53	3.65	7.31	5.18	1.63	1.77	4.42	482
Congenital birth defects	8.04	9.45	27.51	10.16	4.88	7.07	10.75	1173
Digestive diseases	111.75	237.50	178.49	130.71	79.99	219.08	199.17	21 738
Musculoskeletal disorders	23.51	10.78	10.21	14.35	16.08	21.20	12.65	1381
Acute infectious diseases	24.74	15.35	14.29	34.27	21.37	28.27	19.08	2082
Urogenital diseases	34.64	52.04	71.89	65.19	24.63	63.60	52.85	5768
Endocrine and metabolic disorders	88.14	50.78	39.97	38.59	70.63	95.41	52.60	5741
Blood disorders	22.27	14.57	13.22	26.34	28.09	107.77	17.88	1951
Sense organ diseases	0	0.03	0	0	0	0	0.02	2
Diarrheal diseases	4.64	1.97	1.18	2.69	5.09	15.90	2.46	268
Skin diseases	35.67	23.55	6.23	30.20	17.91	8.83	23.75	2592
Maternal disorders	1.86	0.39	3.87	0.46	0.41	0	0.82	90
Mental disorders	49.59	11.17	29.23	14.15	8.75	14.13	16.45	1795
Kidney disease	126.29	92.96	80.92	110.07	126.20	219.08	99.44	10 853
Nutritional deficiencies	6.91	16.79	8.60	23.59	15.67	12.37	16.09	1756
Chronic intermediate and immediate cause	31.65	47.63	32.13	71.41	39.69	28.27	47.75	5212
Cardiovascular diseases	462.27	467.05	553.41	438.31	660.70	818.02	480.51	52 443
Neoplasm	74.33	54.96	153.13	86.03	43.56	100.71	69.12	7544
Diabetes and diabetes-related disorders	69.59	66.53	43.63	71.28	89.76	91.87	66.69	7279
Acute intermediate and immediate cause	609.90	718.44	590.80	748.21	638.92	602.47	697.90	76 169
Neurological disorders	63.40	59.16	54.37	98.47	76.74	40.64	65.32	7129
Chronic respiratory diseases	125.67	79.36	97.79	85.37	77.55	72.44	85.77	9361
HIV	4.23	0.81	4.41	4.00	0.41	1.77	1.85	202
Acute respiratory infections	62.27	51.94	85.54	71.94	46.20	91.87	58.47	6381
Neonatal disorders	6.19	2.28	15.04	11.86	0	15.90	5.02	548
Poisoning and overdoses	6.29	1.25	13.86	2.42	0.61	0	2.90	317

^a^ Both sexes combined.

^b^ Misadventure was defined as events likely to represent medical error, such as accidental laceration or incorrect dosage.

## Discussion

In a comprehensive study of US mortality associated with AEMT, deaths were observed in every state, in every year, and in each age group. From 1990 to 2016, the absolute number of annual deaths from AEMT increased marginally, whereas the age-standardized mortality rate decreased, suggesting the absolute increase may be largely due to population increases. Several states had significant decreases in mortality over time. Increases in mortality associated with AEMT were observed with advancing age and in a higher proportion among very young individuals. Mortality associated with AEMT as either an underlying or a contributing cause appeared in 2.8% of all US deaths.

Consistent with prior studies, an overall increase in AEMT was observed with advancing age.^28,39,40^ The vulnerability seen in elderly individuals has been well demonstrated in several areas, including adverse drug events and postoperative complications.^41,42,43^ The mortality cause fraction was also shown to be disproportionally higher in young individuals (Figure 1). For pediatric patients (particularly the very young and premature), the additional risk for AEMT may stem from myriad unique considerations in children that present additional safety challenges, such as the dosing and administration of medication, as well as the complexity inherent in often rare pediatric surgical conditions. Taken together, this may also reflect the increased prevalence of medical contact in both the very young and old populations, allowing for more opportunity for AEMT to occur. Although the overall number of events was low among the very young population (Figure 3B), the high cause fraction is concerning and should be an area of further investigation.

State-to-state variability in age-standardized death rates from AEMT was observed (Figure 2A). Estimates between top and bottom performers varied up to 2-fold, perhaps reflecting general regional differences in quality and safety of care that have been noted in other reports.^44,45,46^ Variation in health care utilization may be a contributing factor as well. Given that surgical and perioperative AEMT is the most common subtype of AEMT, the southeast region’s higher AEMT mortality estimates may be accounted for by the area’s higher-than-average surgical volume, representing more opportunities for harm to occur.^47^ The interstate variability in this report highlights the need for further understanding on what may drive regional differences. Other potential contributors include variability in state regulatory efforts (eg, mandatory adverse event reporting), information technology adoption, patient and clinician characteristics, staffing patterns including nurse-patient ratios, and the level to which health systems have adopted care standardization practices. Understanding variability in these domains across states represents an important opportunity for states to learn from one another and to assist in state and national efforts to reduce AEMT.

Patients whose underlying cause of death was traumatic injury were the most likely to have AEMT listed in the cause-of-death chain on their death certificate followed by intermediate causes, such as cardiac arrest and sepsis, cardiovascular diseases, and gastrointestinal conditions that frequently require surgery. Adverse effects of medical treatment are also commonly in the cause-of-death chain of patients whose underlying cause of death is heart disease, which is perhaps a reflection of the large number of patients with heart disease, the complex care these patients receive toward the end of life, and the possibility that they are likely poorly suited to tolerate and recover from an AEMT. These findings highlight the crucial importance of investing in safety and quality in settings where urgent, emergent, and complex procedures are performed, particularly for individuals with several comorbid conditions.

### Strengths and Limitations

Measuring patient safety in a comprehensive manner on a national scale is challenging. Available approaches to detect and attribute mortality due to medical harm require trade-offs in generalizability, resource intensity, and detection sensitivity.^48,49^ The biggest strength of the GBD study’s approach is that it includes the use of a standardized, comprehensive, consistently applied framework for quantifying causes of death over time. It produces internally consistent results by ensuring that the sum of all specific causes of death is exactly equal to all-cause mortality. In addition, the GBD study is comprehensive in scope, covering 23 age groups from birth to 95 years or older in both sexes and in all 50 states using primary data from those locations.

The GBD approach for estimating mortality associated with AEMT also has limitations. First, *ICD*-coded death certificates have been shown to have varying degrees of reliability in identifying medical harm.^50,51^ They may have limited ability to distinguish between variation in completeness of death certificate reporting and variation in the occurrence of AEMT events. It is also probable that many deaths involving AEMT are not captured either because of motivated misreporting or unintentional omission. Single institutions may therefore find intensive medical record review to be helpful for quality improvement by more comprehensively assessing death certification practices and capturing temporal relationships and assigning causality between AEMT and death. Second, there is no venue within administrative data to capture the contribution of factors for which there are no *ICD* codes. For example, although the National Quality Forum lists a “death associated with patient elopement for more than four hours” as a serious reportable adverse event,^52^ elopement would not be captured on a death certificate. Contributions from latent failures of health systems, a major focus of patient safety, would likewise not be captured here. Third, the sensitive nature of medical harm may result in “motivated misreporting” or changes to documentation practices to avoid implication.^53^ Fourth, the GBD study’s cause classification system that assigns each death to only a single underlying cause means that some events associated with AEMT may be grouped elsewhere. Such groupings are dependent on which *ICD* code was assigned as the underlying cause. For example, adverse drug events from prescribed opioids leading to death would likely be assigned to the GBD study’s cause of “opioid abuse” (*ICD-10* code, F11) or “accidental poisoning” (*ICD-10* code, T40) based on the mechanism of death, whereas they are included with medical harm in many other studies based on the association with a prescription.^54,55^ Somewhat analogously, nosocomial infections (*ICD-10* code, Y95) are often coassigned with a pathogen or type of infection when responsible for a death, and, because Y95 does not end up as the single underlying cause on such death certificates, they are not classified in the GBD study as AEMT.^56^

Patient safety is and will remain a major priority in the United States health care system, yet gauging progress to date on a national scale has been limited. The results presented here add to the body of work in evaluating AEMT and provide another lens through which to assess the burden of AEMT to further guide prevention efforts. The GBD methodology allows for a regular, comprehensive, and consistent assessment of AEMT within the United States. It may be a particularly powerful tool to assess high-level trends over time and place AEMT in context with other causes of mortality. Further work is needed to understand differences from GBD estimates to other AEMT detection approaches.

## Conclusions

This study showed a modest reduction in the death rates from AEMT in the United States from 1990 to 2016 while also observing increased mortality risk with advancing age and certain geographic locations. The annual GBD study releases may allow for tracking of the burden and trend of AEMT over time. In conjunction with other detection systems, the GBD study may provide an increasingly robust assessment of the burden of AEMT across the United States.

## Notes

Supplement. **eTable 1.** *International Classification of Diseases, Ninth Revision* (*ICD-9*) Codes Included in the GBD 2016 Cause Category of Adverse Effects of Medical Treatment (AEMT) **eTable 2.** *International Statistical Classification of Diseases and Related Health Problems, Tenth Revision* (*ICD-10*) Codes Included in the GBD 2016 Cause Category of Adverse Effects of Medical Treatment (AEMT) **eTable 3.** Selected Cause-of-Death Ensemble Modeling (CODEm) Covariates Used to Generate the Adverse Effects of Medical Harm (AEMT) Models **eTable 4.** Result of Redistribution of Garbage Codes on AEMT as Underlying Cause by Different Nonunderlying Cause by *ICD-9* and *ICD-10* **eTable 5.** Frequency of Causes of Death in the Cause-of-Death (COD) Chain When Adverse Effects of Medical Treatment (AEMT) Is the Underlying Cause of Death, United States 1980-2014 **eTable 6.** Number of Each Subtype of AEMT by Year Where AEMT Was Certified as the Underlying Cause of Death, 1980 to 2014, Both Sexes Combined **eTable 7.** Rate of Occurrence of AEMT in the Cause-of Death Chain by Other Cause Category (per 1000 Deaths in Each Category) When AEMT Was NOT Certified as the Underlying Cause of Death, 1980 to 2014, Both Sexes Combined **eMethods.** **eReferences.**
