# Supplementary material for: Association of Adverse Effects of Medical Treatment With Mortality in the United States: A Secondary Analysis of the Global Burden of Diseases, Injuries, and Risk Factors Study
Source: JAMA Netw Open. 2019 Jan 18;2(1):e187041. doi: 10.1001/jamanetworkopen.2018.7041 (PMC6484545; doi:10.1001/jamanetworkopen.2018.7041)
Supplement: Supplement. — eTable 1. International Classification of Diseases, Ninth Revision (ICD-9) Codes Included in the GBD 2016 Cause Category of Adverse Effects of Medical Treatment (AEMT) eTable 2. International Statistical Classification of Diseases and Related Health Problems, Tenth Revision (ICD-10) Codes Included in the GBD 2016 Cause Category of Adverse Effects of Medical Treatment (AEMT) eTable 3. Selected Cause-of-Death Ensemble Modeling (CODEm) Covariates Used to Generate the Adverse Effects of Medical Harm (AEMT) Models eTable 4. Result of Redistribution of Garbage Codes on AEMT as Underlying Cause by Different Nonunderlying Cause by ICD-9 and ICD-10 eTable 5. Frequency of Causes of Death in the Cause-of-Death (COD) Chain When Adverse Effects of Medical Treatment (AEMT) Is the Underlying Cause of Death, United States 1980-2014 eTable 6. Number of Each Subtype of AEMT by Year Where AEMT Was Certified as the Underlying Cause of Death, 1980 to 2014, Both Sexes Combined eTable 7. Rate of Occurrence of AEMT in the Cause-of Death Chain by Other Cause Category (per 1000 Deaths in Each Category) When AEMT Was NOT Certified as the Underlying Cause of Death, 1980 to 2014, Both Sexes Combined eMethods. eReferences. [file jamanetwopen-2-e187041-s001.pdf]

Supplementary Online Content

Sunshine JE, Meo N, Kassebaum NJ, Collison ML, Mokdad AH, Naghavi M. Association of adverse effects of medical treatment with mortality in the United States: a secondary analysis of the Global Burden of Diseases, Injuries, and Risk Factors Study. *JAMA Netw Open*. 2019; 2(1):e187041. doi:0.1001/jamanetworkopen.2018.7041

eTable 1. *International Classification of Diseases, Ninth Revision* (*ICD-9*) Codes Included in the GBD 2016 Cause Category of Adverse Effects of Medical Treatment (AEMT)

eTable 2. *International Statistical Classification of Diseases and Related Health Problems, Tenth Revision* (*ICD-10*) Codes Included in the GBD 2016 Cause Category of Adverse Effects of Medical Treatment (AEMT)

eTable 3. Selected Cause-of-Death Ensemble Modeling (CODEm) Covariates Used to Generate the Adverse Effects of Medical Harm (AEMT) Models

eTable 4. Result of Redistribution of Garbage Codes on AEMT as Underlying Cause by Different Nonunderlying Cause by *ICD-9* and *ICD-10*

eTable 5. Frequency of Causes of Death in the Cause-of-Death (COD) Chain When Adverse Effects of Medical Treatment (AEMT) Is the Underlying Cause of Death, United States 1980-2014

eTable 6. Number of Each Subtype of AEMT by Year Where AEMT Was Certified as the Underlying Cause of Death, 1980 to 2014, Both Sexes Combined

eTable 7. Rate of Occurrence of AEMT in the Cause-of Death Chain by Other Cause Category (per 1000 Deaths in Each Category) When AEMT Was NOT Certified as the Underlying Cause of Death, 1980 to 2014, Both Sexes Combined

eMethods.

eReferences.

This supplementary material has been provided by the authors to give readers additional information about their work.

© 2019 Sunshine JE et al. *JAMA Network Open*.

eTable 1. *International Classification of Diseases, Ninth Revision* (*ICD-9*) codes included in the GBD 2016 cause category of adverse effects of medical treatment (AEMT)

ICD-9 code	ICD-9 cause name	AEMT sub-category
244.0	Postsurgical hypothyroidism	2 - Surgical and peri-operative adverse events
244.1	Other postablative hypothyroidism	4 - Medical management
244.3	Other iatrogenic hypothyroidism	6 - Other
244.8	Other specified acquired hypothyroidism	6 - Other
251.3	Postsurgical hypoinsulinemia	2 - Surgical and peri-operative adverse events
253.7	Iatrogenic pituitary disorders	6 - Other
331.81	Reye's syndrome	1 - Adverse drug events
349.0	Reaction to spinal or lumbar puncture	4 - Medical management
349.1	Nervous system complications from surgically implanted device	5 - Medical or surgical devices
349.31	Accidental puncture or laceration of dura during a procedure	3 - Misadventure
357.6	Polyneuropathy due to drugs	1 - Adverse drug events
359.24	Drug- induced myotonia	1 - Adverse drug events
457.0	Postmastectomy lymphedema syndrome	2 - Surgical and peri-operative adverse events
518.7	Transfusion related acute lung injury (TRALI)	4 - Medical management
519.0	Tracheostomy	2 - Surgical and peri-operative adverse events
519.00	Tracheostomy complication, unspecified	2 - Surgical and peri-operative adverse events
519.01	Infection of tracheostomy	2 - Surgical and peri-operative adverse events
519.02	Mechanical complication of tracheostomy	2 - Surgical and peri-operative adverse events
519.09	Other tracheostomy complications	2 - Surgical and peri-operative adverse events
536.4	Gastrostomy complications	2 - Surgical and peri-operative adverse events
536.40	Gastrostomy complication, unspecified	2 - Surgical and peri-operative adverse events
536.41	Infection of gastrostomy	2 - Surgical and peri-operative adverse events
536.42	Mechanical complication of gastrostomy	2 - Surgical and peri-operative adverse events
536.49	Other gastrostomy complications	2 - Surgical and peri-operative adverse events
**539**	**Complications of bariatric procedures**	2 - Surgical and peri-operative adverse events
539.0	Complications of gastric band procedure	2 - Surgical and peri-operative adverse events
539.01	Infection due to gastric band procedure	2 - Surgical and peri-operative adverse events
539.09	Other complications of gastric band procedure	2 - Surgical and peri-operative adverse events
539.8	Complications of other bariatric procedure	2 - Surgical and peri-operative adverse events
539.81	Infection due to other bariatric procedure	2 - Surgical and peri-operative adverse events
539.89	Other complications of other bariatric procedure	2 - Surgical and peri-operative adverse events
539.9	Ventral hernia	2 - Surgical and peri-operative adverse events
551.2	Ventral hernia with gangrene	2 - Surgical and peri-operative adverse events
551.20	Ventral hernia, unspecified, with gangrene	2 - Surgical and peri-operative adverse events
551.21	Incisional ventral hernia, with gangrene	2 - Surgical and peri-operative adverse events
551.29	Other ventral hernia with gangrene	2 - Surgical and peri-operative adverse events
552.2	Ventral hernia with obstruction	2 - Surgical and peri-operative adverse events
552.20	Ventral, unspecified, hernia with obstruction	2 - Surgical and peri-operative adverse events
552.21	Incisional ventral hernia with obstruction	2 - Surgical and peri-operative adverse events
552.29	Other ventral hernia with obstruction	2 - Surgical and peri-operative adverse events
564.2	Postgastric surgery syndromes	2 - Surgical and peri-operative adverse events
564.3	Vomiting following gastrointestinal surgery	2 - Surgical and peri-operative adverse events
564.4	Other postoperative functional disorders	2 - Surgical and peri-operative adverse events
569.6	Colostomy and enterostomy complications	2 - Surgical and peri-operative adverse events
569.60	Colostomy and enterostomy complication, unspecified	2 - Surgical and peri-operative adverse events
569.61	Infection of colostomy or enterostomy	2 - Surgical and peri-operative adverse events
569.62	Mechanical complication of colostomy and enterostomy	2 - Surgical and peri-operative adverse events
569.69	Other colostomy and enterostomy complication	2 - Surgical and peri-operative adverse events
579.3	Other and unspecified postsurgical nonabsorption	2 - Surgical and peri-operative adverse events
596.82	Mechanical complication of cystostomy	2 - Surgical and peri-operative adverse events
596.83	Other complication of cystostomy	2 - Surgical and peri-operative adverse events
598.2	Postoperative urethral stricture	2 - Surgical and peri-operative adverse events
779.4	Drug reactions and intoxications specific to newborn	1 - Adverse drug events
779.5	Drug withdrawal syndrome in newborn	1 - Adverse drug events
780.62	Postprocedural fever	2 - Surgical and peri-operative adverse events
780.63	Postvaccination fever	1 - Adverse drug events

© 2019 Sunshine JE et al. *JAMA Network Open*.

**E870**	**Accidental cut puncture perforation or hemorrhage during medical care**	3 - Misadventure
E870.0	Accidental cut, puncture, perforation or hemorrhage during surgical operation	3 - Misadventure
E870.1	Accidental cut, puncture, perforation or hemorrhage during infusion or transfusion	3 - Misadventure
E870.2	Accidental cut, puncture, perforation or hemorrhage during kidney dialysis or other perfusion	3 - Misadventure
E870.3	Accidental cut, puncture, perforation or hemorrhage during injection or vaccination	3 - Misadventure
E870.4	Accidental cut, puncture, perforation or hemorrhage during endoscopic examination	3 - Misadventure
E870.5	Accidental cut, puncture, perforation or hemorrhage during aspiration of fluid or tissue, puncture, and catheterization	3 - Misadventure
E870.6	Accidental cut, puncture, perforation or hemorrhage during heart catheterization	3 - Misadventure
E870.7	Accidental cut, puncture, perforation or hemorrhage during administration of enema	3 - Misadventure
E870.8	Accidental cut, puncture, perforation or hemorrhage during other specified medical care	3 - Misadventure
E870.9	Accidental cut, puncture, perforation or hemorrhage during unspecified medical care	3 - Misadventure
**E871**	**Foreign object left in body during procedure**	3 - Misadventure
E871.0	Foreign object left in body during surgical operation	3 - Misadventure
E871.1	Foreign object left in body during infusion or transfusion	3 - Misadventure
E871.2	Foreign object left in body during kidney dialysis or other perfusion	3 - Misadventure
E871.3	Foreign object left in body during injection or vaccination	3 - Misadventure
E871.4	Foreign object left in body during endoscopic examination	3 - Misadventure
E871.5	Foreign object left in body during aspiration of fluid or tissue, puncture, and catheterization	3 - Misadventure
E871.6	Foreign object left in body during heart catheterization	3 - Misadventure
E871.7	Foreign object left in body during removal of catheter or packing	3 - Misadventure
E871.8	Foreign object left in body during other specified procedures	3 - Misadventure
E871.9	Foreign object left in body during unspecified procedure	3 - Misadventure
**E872**	**Failure of sterile precautions during procedure**	3 - Misadventure
E872.0	Failure of sterile precautions during surgical operation	3 - Misadventure
E872.1	Failure of sterile precautions during infusion or transfusion	3 - Misadventure
E872.2	Failure of sterile precautions during kidney dialysis and other perfusion	3 - Misadventure
E872.3	Failure of sterile precautions during injection or vaccination	3 - Misadventure
E872.4	Failure of sterile precautions during endoscopic examination	3 - Misadventure
E872.5	Failure of sterile precautions during aspiration of fluid or tissue, puncture, and catheterization	3 - Misadventure
E872.6	Failure of sterile precautions during heart catheterization	3 - Misadventure
E872.8	Failure of sterile precautions during other specified procedures	3 - Misadventure
E872.9	Failure of sterile precautions during unspecified procedure	3 - Misadventure
**E873**	**Failure in dosage**	3 - Misadventure
E873.0	Excessive amount of blood or other fluid during transfusion or infusion	3 - Misadventure
E873.1	Incorrect dilution of fluid during infusion	3 - Misadventure
E873.2	Overdose of radiation in therapy	3 - Misadventure
E873.3	Inadvertent exposure of patient to radiation during medical care	3 - Misadventure
E873.4	Failure in dosage in electroshock or insulin-shock therapy	3 - Misadventure
E873.5	Inappropriate [too hot or too cold] temperature in local application and packing	3 - Misadventure
E873.6	Nonadministration of necessary drug or medicinal substance	3 - Misadventure
E873.8	Other specified failure in dosage	3 - Misadventure
E873.9	Unspecified failure in dosage	3 - Misadventure
**E874**	**Mechanical failure of instrument or apparatus during procedure**	3 - Misadventure
E874.0	Mechanical failure of instrument or apparatus during surgical operation	3 - Misadventure
E874.1	Mechanical failure of instrument or apparatus during infusion and transfusion	3 - Misadventure
E874.2	Mechanical failure of instrument or apparatus during kidney dialysis and other perfusion	3 - Misadventure
E874.3	Mechanical failure of instrument or apparatus during endoscopic examination	3 - Misadventure
E874.4	Mechanical failure of instrument or apparatus during aspiration of fluid or tissue, puncture, and catheterization	3 - Misadventure
E874.5	Mechanical failure of instrument or apparatus during heart catheterization	3 - Misadventure
E874.8	Mechanical failure of instrument or apparatus during other specified procedures	3 - Misadventure
E874.9	Mechanical failure of instrument or apparatus during unspecified procedure	3 - Misadventure
**E875**	**Contaminated or infected blood other fluid drug or biological substance**	3 - Misadventure
E875.0	Contaminated substance transfused or infused	3 - Misadventure
E875.1	Contaminated substance injected or used for vaccination	3 - Misadventure
E875.2	Contaminated drug or biological substance administered by other means	3 - Misadventure

© 2019 Sunshine JE et al. *JAMA Network Open*.

E875.8	Misadventure to patient from other contamination	3 - Misadventure
E875.9	Misadventure to patient from unspecified contamination	3 - Misadventure
**E876**	**Other and unspecified misadventures during medical care**	3 - Misadventure
E876.0	Mismatched blood in transfusion	3 - Misadventure
E876.1	Wrong fluid in infusion	3 - Misadventure
E876.2	Failure in suture and ligature during surgical operation	3 - Misadventure
E876.3	Endotracheal tube wrongly placed during anesthetic procedure	3 - Misadventure
E876.4	Failure to introduce or to remove other tube or instrument	3 - Misadventure
E876.5	Performance of wrong operation (procedure) on correct patient	3 - Misadventure
E876.6	Performance of operation (procedure) on patient not scheduled for surgery	3 - Misadventure
E876.7	Performance of correct operation (procedure) on wrong side/body part	3 - Misadventure
E876.8	Other specified misadventures during medical care	3 - Misadventure
E876.9	Unspecified misadventure during medical care	3 - Misadventure
**E878**	**Surgical operation and other surgical procedures as the cause of abnormal reaction of patient or of later complication without mention of misadventure at the time of operation**	2 - Surgical and peri-operative adverse events
E878.0	Surgical operation with transplant of whole organ causing abnormal patient reaction, or later complication, without mention of misadventure at time of operation	2 - Surgical and peri-operative adverse events
E878.1	Surgical operation with implant of artificial internal device causing abnormal patient reaction, or later complication,without mention of misadventure at time of operation	5 - Medical or surgical devices
E878.2	Surgical operation with anastomosis, bypass, or graft, with natural or artificial tissues used as implant causing abnormal patient reaction, or later complication, without mention of misadventure at time of operation	2 - Surgical and peri-operative adverse events
E878.3	Surgical operation with formation of external stoma causing abnormal patient reaction, or later complication, without mention of misadventure at time of operation	2 - Surgical and peri-operative adverse events
E878.4	Other restorative surgery causing abnormal patient reaction, or later complication, without mention of misadventure at time of operation	2 - Surgical and peri-operative adverse events
E878.5	Amputation of limb(s) causing abnormal patient reaction, or later complication, without mention of misadventure at time of operation	2 - Surgical and peri-operative adverse events
E878.6	Removal of other organ (partial) (total) causing abnormal patient reaction, or later complication, without mention of misadventure at time of operation	2 - Surgical and peri-operative adverse events
E878.8	Other specified surgical operations and procedures causing abnormal patient reaction, or later complication, without mention of misadventure at time of operation	2 - Surgical and peri-operative adverse events
E878.9	Unspecified surgical operations and procedures causing abnormal patient reaction, or later complication, without mention of misadventure at time of operation	2 - Surgical and peri-operative adverse events
**E879**	**Other procedures without mention of misadventure at the time of procedure as the cause of abnormal reaction of patient or of later complication**	4 - Medical management
E879.0	Cardiac catheterization as the cause of abnormal reaction of patient, or of later complication, without mention of misadventure at time of procedure	2 - Surgical and peri-operative adverse events
E879.1	Kidney dialysis as the cause of abnormal reaction of patient, or of later complication, without mention of misadventure at time of procedure	4 - Medical management
E879.2	Radiological procedure and radiotherapy as the cause of abnormal reaction of patient, or of later complication, without mention of misadventure at time of procedure	4 - Medical management
E879.3	Shock therapy as the cause of abnormal reaction of patient, or of later complication, without mention of misadventure at time of procedure	4 - Medical management
E879.4	Aspiration of fluid as the cause of abnormal reaction of patient, or of later complication, without mention of misadventure at time of procedure	4 - Medical management
E879.5	Insertion of gastric or duodenal sound as the cause of abnormal reaction of patient, or of later complication, without mention of misadventure of time of procedure	4 - Medical management
E879.6	Urinary catheterization as the cause of abnormal reaction of patient, or of later complication, without mention of misadventure at time of procedure	4 - Medical management
E879.7	Blood sampling as the cause of abnormal reaction of patient, or of later complication, without mention of misadventure at time of procedure	4 - Medical management
E879.8	Other specified procedures as the cause of abnormal reaction of patient, or of later complication, without mention of misadventure at time of procedure	4 - Medical management
E879.9	Unspecified procedure as the cause of abnormal reaction of patient, or of later complication, without mention of misadventure at time of procedure	4 - Medical management
**E930**	**Antibiotics causing adverse effects in therapeutic use**	1 - Adverse drug events
E930.0	Penicillins causing adverse effects in therapeutic use	1 - Adverse drug events
E930.1	Antifungal antibiotics causing adverse effects in therapeutic use	1 - Adverse drug events
E930.2	Chloramphenicol group causing adverse effects in therapeutic use	1 - Adverse drug events
E930.3	Erythromycin and other macrolides causing adverse effects in therapeutic use	1 - Adverse drug events
E930.4	Tetracycline group causing adverse effects in therapeutic use	1 - Adverse drug events

© 2019 Sunshine JE et al. *JAMA Network Open*.

E930.5	Cephalosporin group causing adverse effects in therapeutic use	1 - Adverse drug events
E930.6	Antimycobacterial antibiotics causing adverse effects in therapeutic use	1 - Adverse drug events
E930.7	Antineoplastic antibiotics causing adverse effects in therapeutic use	1 - Adverse drug events
E930.8	Other specified antibiotics causing adverse effects in therapeutic use	1 - Adverse drug events
E930.9	Unspecified antibiotic causing adverse effects in therapeutic use	1 - Adverse drug events
**E931**	**Other anti-infectives causing adverse effects in therapeutic use**	1 - Adverse drug events
E931.0	Sulfonamides causing adverse effects in therapeutic use	1 - Adverse drug events
E931.1	Arsenical anti-infectives causing adverse effects in therapeutic use	1 - Adverse drug events
E931.2	Heavy metal anti-infectives causing adverse effects in therapeutic use	1 - Adverse drug events
E931.3	Quinoline and hydroxyquinoline derivatives causing adverse effects in therapeutic use	1 - Adverse drug events
E931.4	Antimalarials and drugs acting on other blood protozoa causing adverse effects in therapeutic use	1 - Adverse drug events
E931.5	Other antiprotozoal drugs causing adverse effects in therapeutic use	1 - Adverse drug events
E931.6	Anthelmintics causing adverse effects in therapeutic use	1 - Adverse drug events
E931.7	Antiviral drugs causing adverse effects in therapeutic use	1 - Adverse drug events
E931.8	Other antimycobacterial drugs causing adverse effects in therapeutic use	1 - Adverse drug events
E931.9	Other and unspecified anti-infectives causing adverse effects in therapeutic use	1 - Adverse drug events
**E932**	**Hormones and synthetic substitutes causing adverse effects in therapeutic use**	1 - Adverse drug events
E932.0	Adrenal cortical steroids causing adverse effects in therapeutic use	1 - Adverse drug events
E932.1	Androgens and anabolic congeners causing adverse effects in therapeutic use	1 - Adverse drug events
E932.2	Ovarian hormones and synthetic substitutes causing adverse effects in therapeutic use	1 - Adverse drug events
E932.3	Insulins and antidiabetic agents causing adverse effects in therapeutic use	1 - Adverse drug events
E932.4	Anterior pituitary hormones causing adverse effects in therapeutic use	1 - Adverse drug events
E932.5	Posterior pituitary hormones causing adverse effects in therapeutic use	1 - Adverse drug events
E932.6	Parathyroid and parathyroid derivatives causing adverse effects in therapeutic use	1 - Adverse drug events
E932.7	Thyroid and thyroid derivatives causing adverse effects in therapeutic use	1 - Adverse drug events
E932.8	Antithyroid agents causing adverse effects in therapeutic use	1 - Adverse drug events
E932.9	Other and unspecified hormones and synthetic substitutes causing adverse effects in therapeutic use	1 - Adverse drug events
**E933**	**Primarily systemic agents causing adverse effects in therapeutic use**	1 - Adverse drug events
E933.0	Antiallergic and antiemetic drugs causing adverse effects in therapeutic use	1 - Adverse drug events
E933.1	Antineoplastic and immunosuppressive drugs causing adverse effects in therapeutic use	1 - Adverse drug events
E933.2	Acidifying agents causing adverse effects in therapeutic use	1 - Adverse drug events
E933.3	Alkalizing agents causing adverse effects in therapeutic use	1 - Adverse drug events
E933.4	Enzymes, not elsewhere classified, causing adverse effects in therapeutic use	1 - Adverse drug events
E933.5	Vitamins, not elsewhere classified, causing adverse effects in therapeutic use	1 - Adverse drug events
E933.6	Oral bisphosphonates	1 - Adverse drug events
E933.7	Intravenous bisphosphonates	1 - Adverse drug events
E933.8	Other systemic agents, not elsewhere classified, causing adverse effects in therapeutic use	1 - Adverse drug events
E933.9	Unspecified systemic agent causing adverse effects in therapeutic use	1 - Adverse drug events
**E934**	**Agents primarily affecting blood constituents causing adverse effects in therapeutic use**	1 - Adverse drug events
E934.0	Iron and its compounds causing adverse effects in therapeutic use	1 - Adverse drug events
E934.1	Liver preparations and other antianemic agents causing adverse effects in therapeutic use	1 - Adverse drug events
E934.2	Anticoagulants causing adverse effects in therapeutic use	1 - Adverse drug events
E934.3	Vitamin k [phytonadione] causing adverse effects in therapeutic use	1 - Adverse drug events
E934.4	Fibrinolysis-affecting drugs causing adverse effects in therapeutic use	1 - Adverse drug events
E934.5	Anticoagulant antagonists and other coagulants causing adverse effects in therapeutic use	1 - Adverse drug events
E934.6	Gamma globulin causing adverse effects in therapeutic use	1 - Adverse drug events
E934.7	Natural blood and blood products causing adverse effects in therapeutic use	1 - Adverse drug events
E934.8	Other agents affecting blood constituents causing adverse effects in therapeutic use	1 - Adverse drug events
E934.9	Unspecified agent affecting blood constituents causing adverse effects in therapeutic use	1 - Adverse drug events
**E935**	**Analgesics antipyretics and antirheumatics causing adverse effects in therapeutic use**	1 - Adverse drug events
E935.0	Heroin causing adverse effects in therapeutic use	1 - Adverse drug events
E935.1	Methadone causing averse effects in therapeutic use	1 - Adverse drug events
E935.2	Other opiates and related narcotics causing adverse effects in therapeutic use	1 - Adverse drug events
E935.3	Salicylates causing adverse effects in therapeutic use	1 - Adverse drug events

© 2019 Sunshine JE et al. *JAMA Network Open*.

E935.4	Aromatic analgesics, not elsewhere classified, causing adverse effects in therapeutic use	1 - Adverse drug events
E935.5	Pyrazole derivatives causing adverse effects in therapeutic use	1 - Adverse drug events
E935.6	Antirheumatics [antiphlogistics] causing adverse effects in therapeutic use	1 - Adverse drug events
E935.7	Other non-narcotic analgesics causing adverse effects in therapeutic use	1 - Adverse drug events
E935.8	Other specified analgesics and antipyretics causing adverse effects in therapeutic use	1 - Adverse drug events
E935.9	Unspecified analgesic and antipyretic causing adverse effects in therapeutic use	1 - Adverse drug events
**E936**	**Anticonvulsants and anti-parkinsonism drugs causing adverse effects in therapeutic use**	1 - Adverse drug events
E936.0	Oxazolidine derivatives causing adverse effects in therapeutic use	1 - Adverse drug events
E936.1	Hydantoin derivatives causing adverse effects in therapeutic use	1 - Adverse drug events
E936.2	Succinimides causing adverse effects in therapeutic use	1 - Adverse drug events
E936.3	Other and unspecified anticonvulsants causing adverse effects in therapeutic use	1 - Adverse drug events
E936.4	Anti-parkinsonism drugs causing adverse effects in therapeutic use	1 - Adverse drug events
**E937**	**Sedatives and hypnotics causing adverse effects in therapeutic use**	1 - Adverse drug events
E937.0	Barbiturates causing adverse effects in therapeutic use	1 - Adverse drug events
E937.1	Chloral hydrate group causing adverse effects in therapeutic use	1 - Adverse drug events
E937.2	Paraldehyde causing adverse effects in therapeutic use	1 - Adverse drug events
E937.3	Bromine compounds causing adverse effects in therapeutic use	1 - Adverse drug events
E937.4	Methaqualone compounds causing adverse effects in therapeutic use	1 - Adverse drug events
E937.5	Glutethimide group causing adverse effects in therapeutic use	1 - Adverse drug events
E937.6	Mixed sedatives, not elsewhere classified, causing adverse effects in therapeutic use	1 - Adverse drug events
E937.8	Other sedatives and hypnotics causing adverse effects in therapeutic use	1 - Adverse drug events
E937.9	Unspecified sedatives and hypnotics causing adverse effects in therapeutic use	1 - Adverse drug events
**E938**	**Other central nervous system depressants and anesthetics causing adverse effects in therapeutic use**	1 - Adverse drug events
E938.0	Central nervous system muscle-tone depressants causing adverse effects in therapeutic use	2 - Surgical and peri-operative adverse events
E938.1	Halothane causing adverse effects in therapeutic use	2 - Surgical and peri-operative adverse events
E938.2	Other gaseous anesthetics causing adverse effects in therapeutic use	2 - Surgical and peri-operative adverse events
E938.3	Intravenous anesthetics causing adverse effects in therapeutic use	2 - Surgical and peri-operative adverse events
E938.4	Other and unspecified general anesthetics causing adverse effects in therapeutic use	2 - Surgical and peri-operative adverse events
E938.5	Surface and infiltration anesthetics causing adverse effects in therapeutic use	2 - Surgical and peri-operative adverse events
E938.6	Peripheral nerve- and plexus-blocking anesthetics causing adverse effects in therapeutic use	2 - Surgical and peri-operative adverse events
E938.7	Spinal anesthetics causing adverse effects in therapeutic use	2 - Surgical and peri-operative adverse events
E938.9	Other and unspecified local anesthetics causing adverse effects in therapeutic use	2 - Surgical and peri-operative adverse events
**E939**	**Psychotropic agents causing adverse effects in therapeutic use**	1 - Adverse drug events
E939.0	Antidepressants causing adverse effects in therapeutic use	1 - Adverse drug events
E939.1	Phenothiazine-based tranquilizers causing adverse effects in therapeutic use	1 - Adverse drug events
E939.2	Butyrophenone-based tranquilizers causing adverse effects in therapeutic use	1 - Adverse drug events
E939.3	Other antipsychotics, neuroleptics, and major tranquilizers causing adverse effects in therapeutic use	1 - Adverse drug events
E939.4	Benzodiazepine-based tranquilizers causing adverse effects in therapeutic use	1 - Adverse drug events
E939.5	Other tranquilizers causing adverse effects in therapeutic use	1 - Adverse drug events
E939.6	Psychodysleptics [hallucinogens] causing adverse effects in therapeutic use	1 - Adverse drug events
E939.7	Psychostimulants causing adverse effects in therapeutic use	1 - Adverse drug events
E939.8	Other psychotropic agents causing adverse effects in therapeutic use	1 - Adverse drug events
E939.9	Unspecified psychotropic agent causing adverse effects in therapeutic use	1 - Adverse drug events
**E940**	**Central nervous system stimulants causing adverse effects in therapeutic use**	1 - Adverse drug events
E940.0	Analeptics causing adverse effects in therapeutic use	1 - Adverse drug events
E940.1	Opiate antagonists causing adverse effects in therapeutic use	1 - Adverse drug events
E940.8	Other specified central nervous system stimulants causing adverse effects in therapeutic use	1 - Adverse drug events
E940.9	Unspecified central nervous system stimulant causing adverse effects in therapeutic use	1 - Adverse drug events
**E941**	**Drugs primarily affecting the autonomic nervous system causing adverse effects in therapeutic use**	1 - Adverse drug events
E941.0	Parasympathomimetics [cholinergics] causing adverse effects in therapeutic use	1 - Adverse drug events
E941.1	Parasympatholytics [anticholinergics and antimuscarinics] and spasmolytics causing adverse effects in therapeutic use	1 - Adverse drug events
E941.2	Sympathomimetics [adrenergics] causing adverse effects in therapeutic use	1 - Adverse drug events

© 2019 Sunshine JE et al. *JAMA Network Open*.

E941.3	Sympatholytics [antiadrenergics] causing adverse effects in therapeutic use	1 - Adverse drug events
E941.9	Unspecified drug primarily affecting the autonomic nervous system causing adverse effects in therapeutic use	1 - Adverse drug events
**E942**	**Agents primarily affecting the cardiovascular system causing adverse effects in therapeutic use**	1 - Adverse drug events
E942.0	Cardiac rhythm regulators causing adverse effects in therapeutic use	1 - Adverse drug events
E942.1	Cardiotonic glycosides and drugs of similar action causing adverse effects in therapeutic use	1 - Adverse drug events
E942.2	Antilipemic and antiarteriosclerotic drugs causing adverse effects in therapeutic use	1 - Adverse drug events
E942.3	Ganglion-blocking agents causing adverse effects in therapeutic use	1 - Adverse drug events
E942.4	Coronary vasodilators causing adverse effects in therapeutic use	1 - Adverse drug events
E942.5	Other vasodilators causing adverse effects in therapeutic use	1 - Adverse drug events
E942.6	Other antihypertensive agents causing adverse effects in therapeutic use	1 - Adverse drug events
E942.7	Antivaricose drugs, including sclerosing agents, causing adverse effects in therapeutic use	1 - Adverse drug events
E942.8	Capillary-active drugs causing adverse effects in therapeutic use	1 - Adverse drug events
E942.9	Other and unspecified agents primarily affecting the cardiovascular system causing adverse effects in therapeutic use	1 - Adverse drug events
**E943**	**Agents primarily affecting gastrointestinal system causing adverse effects in therapeutic use**	1 - Adverse drug events
E943.0	Antacids and antigastric secretion drugs causing adverse effects in therapeutic use	1 - Adverse drug events
E943.1	Irritant cathartics causing adverse effects in therapeutic use	1 - Adverse drug events
E943.2	Emollient cathartics causing adverse effects in therapeutic use	1 - Adverse drug events
E943.3	Other cathartics, including intestinal atonia drugs, causing adverse effects in therapeutic use	1 - Adverse drug events
E943.4	Digestants causing adverse effects in therapeutic use	1 - Adverse drug events
E943.5	Antidiarrheal drugs causing adverse effects in therapeutic use	1 - Adverse drug events
E943.6	Emetics causing adverse effects in therapeutic use	1 - Adverse drug events
E943.8	Other specified agents primarily affecting the gastro-intestinal system causing adverse effects in therapeutic use	1 - Adverse drug events
E943.9	Unspecified agent primarily affecting the gastrointestinal system causing adverse effects in therapeutic use	1 - Adverse drug events
**E944**	**Water mineral and uric acid metabolism drugs causing adverse effects in therapeutic use**	1 - Adverse drug events
E944.0	Mercurial diuretics causing adverse effects in therapeutic use	1 - Adverse drug events
E944.1	Purine derivative diuretics causing adverse effects in therapeutic use	1 - Adverse drug events
E944.2	Carbonic acid anhydrase inhibitors causing adverse effects in therapeutic use	1 - Adverse drug events
E944.3	Saluretics causing adverse effects in therapeutic use	1 - Adverse drug events
E944.4	Other diuretics causing adverse effects in therapeutic use	1 - Adverse drug events
E944.5	Electrolytic, caloric, and water-balance agents causing adverse effects in therapeutic use	1 - Adverse drug events
E944.6	Other mineral salts, not elsewhere classified, causing adverse effects in therapeutic use	1 - Adverse drug events
E944.7	Uric acid metabolism drugs causing adverse effects in therapeutic use	1 - Adverse drug events
**E945**	**Agents primarily acting on the smooth and skeletal muscles and respiratory system causing adverse effects in therapeutic use**	1 - Adverse drug events
E945.0	Oxytocic agents causing adverse effects in therapeutic use	1 - Adverse drug events
E945.1	Smooth muscle relaxants causing adverse effects in therapeutic use	1 - Adverse drug events
E945.2	Skeletal muscle relaxants causing adverse effects in therapeutic use	1 - Adverse drug events
E945.3	Other and unspecified drugs acting on muscles causing adverse effects in therapeutic use	1 - Adverse drug events
E945.4	Antitussives causing adverse effects in therapeutic use	1 - Adverse drug events
E945.5	Expectorants causing adverse effects in therapeutic use	1 - Adverse drug events
E945.6	Anti-common cold drugs causing adverse effects in therapeutic use	1 - Adverse drug events
E945.7	Antiasthmatics causing adverse effects in therapeutic use	1 - Adverse drug events
E945.8	Other and unspecified respiratory drugs causing adverse effects in therapeutic use	1 - Adverse drug events
**E946**	**Agents primarily affecting skin and mucous membrane ophthalmological otorhinolaryngological and dental drugs causing adverse effects in therapeutic use**	1 - Adverse drug events
E946.0	Local anti-infectives and anti-inflammatory drugs causing adverse effects in therapeutic use	1 - Adverse drug events
E946.1	Antipruritics causing adverse effects in therapeutic use	1 - Adverse drug events
E946.2	Local astringents and local detergents causing adverse effects in therapeutic use	1 - Adverse drug events
E946.3	Emollients, demulcents, and protectants causing adverse effects in therapeutic use	1 - Adverse drug events

© 2019 Sunshine JE et al. *JAMA Network Open*.

E946.4	Keratolytics, keratoplastics, other hair treatment drugs and preparations causing adverse effects in therapeutic use	1 - Adverse drug events
E946.5	Eye anti-infectives and other eye drugs causing adverse effects in therapeutic use	1 - Adverse drug events
E946.6	Anti-infectives and other drugs and preparations for ear, nose, and throat causing adverse effects in therapeutic use	1 - Adverse drug events
E946.7	Dental drugs topically applied causing adverse effects in therapeutic use	1 - Adverse drug events
E946.8	Other agents primarily affecting skin and mucous membrane causing adverse effects in therapeutic use	1 - Adverse drug events
E946.9	Unspecified agent primarily affecting skin and mucous membrane causing adverse effects in therapeutic use	1 - Adverse drug events
**E947**	**Other and unspecified drugs and medicinal substances causing adverse effects in therapeutic use**	1 - Adverse drug events
E947.0	Dietetics causing adverse effects in therapeutic use	1 - Adverse drug events
E947.1	Lipotropic drugs causing adverse effects in therapeutic use	1 - Adverse drug events
E947.2	Antidotes and chelating agents, not elsewhere classified, causing adverse effects in therapeutic use	1 - Adverse drug events
E947.3	Alcohol deterrents causing adverse effects in therapeutic use	1 - Adverse drug events
E947.4	Pharmaceutical excipients causing adverse effects in therapeutic use	1 - Adverse drug events
E947.8	Other drugs and medicinal substances causing adverse effects in therapeutic use	1 - Adverse drug events
E947.9	Unspecified drug or medicinal substance causing adverse effects in therapeutic use	1 - Adverse drug events
**E948**	**Bacterial vaccines causing adverse effects in therapeutic use**	1 - Adverse drug events
E948.0	Bcg vaccine causing adverse effects in therapeutic use	1 - Adverse drug events
E948.1	Typhoid and paratyphoid vaccines causing adverse effects in therapeutic use	1 - Adverse drug events
E948.2	Cholera vaccine causing adverse effects in therapeutic use	1 - Adverse drug events
E948.3	Plague vaccine causing adverse effects in therapeutic use	1 - Adverse drug events
E948.4	Tetanus vaccine causing adverse effects in therapeutic use	1 - Adverse drug events
E948.5	Diphtheria vaccine causing adverse effects in therapeutic use	1 - Adverse drug events
E948.6	Pertussis vaccine, including combinations with a pertussis component, causing adverse effects in therapeutic use	1 - Adverse drug events
E948.8	Other and unspecified bacterial vaccines causing adverse effects in therapeutic use	1 - Adverse drug events
E948.9	Mixed bacterial vaccines, except combinations with a pertussis component, causing adverse effects in therapeutic use	1 - Adverse drug events
**E949**	**Other vaccines and biological substances causing adverse effects in therapeutic use**	1 - Adverse drug events
E949.0	Smallpox vaccine causing adverse effects in therapeutic use	1 - Adverse drug events
E949.1	Rabies vaccine causing adverse effects in therapeutic use	1 - Adverse drug events
E949.2	Typhus vaccine causing adverse effects in therapeutic use	1 - Adverse drug events
E949.3	Yellow fever vaccine causing adverse effects in therapeutic use	1 - Adverse drug events
E949.4	Measles vaccine causing adverse effects in therapeutic use	1 - Adverse drug events
E949.5	Poliomyelitis vaccine causing adverse effects in therapeutic use	1 - Adverse drug events
E949.6	Other and unspecified viral and rickettsial vaccines causing adverse effects in therapeutic use	1 - Adverse drug events
E949.7	Mixed viral-rickettsial and bacterial vaccines, except combinations with a pertussis component, causing adverse effects in therapeutic use	1 - Adverse drug events
E949.9	Other and unspecified vaccines and biological substances causing adverse effects in therapeutic use	1 - Adverse drug events

© 2019 Sunshine JE et al. *JAMA Network Open*.

eTable 2. *International Statistical Classification of Diseases and Related Health Problems, Tenth Revision* (*ICD-10*) codes included in the GBD 2016 cause category of adverse effects of medical treatment (AEMT)

ICD-10 code	ICD-10 cause name	AEMT sub-category
D52.1	Drug-induced folate deficiency anemia	1 - Adverse drug events
D59.0	Drug-induced autoimmune hemolytic anemia	1 - Adverse drug events
D59.2	Drug-induced nonautoimmune hemolytic anemia	1 - Adverse drug events
D59.6	Hemoglobinuria due to hemolysis from other external causes	6 - Other
D69.5	Secondary thrombocytopenia	6 - Other
D69.51	Posttransfusion purpura	4 - Medical management
D69.59	Other secondary thrombocytopenia	6 - Other
**D78**	**Intraoperative and postprocedural complications of the spleen**	**2 - Surgical and peri-operative adverse events**
D78.0	Intraoperative hemorrhage and hematoma of the spleen complicating a procedure	2 - Surgical and peri-operative adverse events
D78.01	Intraoperative hemorrhage and hematoma of the spleen complicating a procedure on the spleen	2 - Surgical and peri-operative adverse events
D78.02	Intraoperative hemorrhage and hematoma of the spleen complicating other procedure	2 - Surgical and peri-operative adverse events
D78.1	Accidental puncture and laceration of the spleen during a procedure	3 - Misadventure
D78.11	Accidental puncture and laceration of the spleen during a procedure on the spleen	3 - Misadventure
D78.12	Accidental puncture and laceration of the spleen during other procedure	3 - Misadventure
D78.2	Postprocedural hemorrhage and hematoma of the spleen following a procedure	2 - Surgical and peri-operative adverse events
D78.21	Postprocedural hemorrhage and hematoma of the spleen following a procedure on the spleen	2 - Surgical and peri-operative adverse events
D78.22	Postprocedural hemorrhage and hematoma of the spleen following other procedure	2 - Surgical and peri-operative adverse events
D78.8	Other intraoperative and postprocedural complications of the spleen	2 - Surgical and peri-operative adverse events
D78.81	Other intraoperative complications of the spleen	2 - Surgical and peri-operative adverse events
D78.89	Other postprocedural complications of the spleen	2 - Surgical and peri-operative adverse events
E03.2	Hypothyroidism due to medicaments and other exogenous substances	1 - Adverse drug events
E06.4	Drug-induced thyroiditis	1 - Adverse drug events
**E09**	**Drug or chemical induced diabetes mellitus**	1 - Adverse drug events
E09.0	Drug or chemical induced diabetes mellitus with hyperosmolarity	1 - Adverse drug events
E09.00	Drug or chemical induced diabetes mellitus with hyperosmolarity without nonketotic hyperglycemic-hyperosmolar coma (NKHHC)	1 - Adverse drug events
E09.01	Drug or chemical induced diabetes mellitus with hyperosmolarity with coma	1 - Adverse drug events
E09.1	Drug or chemical induced diabetes mellitus with ketoacidosis	1 - Adverse drug events
E09.10	Drug or chemical induced diabetes mellitus with ketoacidosis without coma	1 - Adverse drug events
E09.11	Drug or chemical induced diabetes mellitus with ketoacidosis with coma	1 - Adverse drug events
E09.2	Drug or chemical induced diabetes mellitus with kidney complications	1 - Adverse drug events
E09.21	Drug or chemical induced diabetes mellitus with diabetic nephropathy	1 - Adverse drug events
E09.22	Drug or chemical induced diabetes mellitus with diabetic chronic kidney disease	1 - Adverse drug events
E09.29	Drug or chemical induced diabetes mellitus with other diabetic kidney complication	1 - Adverse drug events
E09.3	Drug or chemical induced diabetes mellitus with ophthalmic complications	1 - Adverse drug events
E09.31	Drug or chemical induced diabetes mellitus with unspecified diabetic retinopathy	1 - Adverse drug events
E09.311	Drug or chemical induced diabetes mellitus with unspecified diabetic retinopathy with macular edema	1 - Adverse drug events
E09.319	Drug or chemical induced diabetes mellitus with unspecified diabetic retinopathy without macular edema	1 - Adverse drug events
E09.32	Drug or chemical induced diabetes mellitus with mild nonproliferative diabetic retinopathy	1 - Adverse drug events
E09.321	Drug or chemical induced diabetes mellitus with mild nonproliferative diabetic retinopathy with macular edema	1 - Adverse drug events
E09.329	Drug or chemical induced diabetes mellitus with mild nonproliferative diabetic retinopathy without macular edema	1 - Adverse drug events
E09.33	Drug or chemical induced diabetes mellitus with moderate nonproliferative diabetic retinopathy	1 - Adverse drug events

© 2019 Sunshine JE et al. *JAMA Network Open*.

E09.331	Drug or chemical induced diabetes mellitus with moderate nonproliferative diabetic retinopathy with macular edema	1 - Adverse drug events
E09.339	Drug or chemical induced diabetes mellitus with moderate nonproliferative diabetic retinopathy without macular edema	1 - Adverse drug events
E09.34	Drug or chemical induced diabetes mellitus with severe nonproliferative diabetic retinopathy	1 - Adverse drug events
E09.341	Drug or chemical induced diabetes mellitus with severe nonproliferative diabetic retinopathy with macular edema	1 - Adverse drug events
E09.349	Drug or chemical induced diabetes mellitus with severe nonproliferative diabetic retinopathy without macular edema	1 - Adverse drug events
E09.35	Drug or chemical induced diabetes mellitus with proliferative diabetic retinopathy	1 - Adverse drug events
E09.351	Drug or chemical induced diabetes mellitus with proliferative diabetic retinopathy with macular edema	1 - Adverse drug events
E09.359	Drug or chemical induced diabetes mellitus with proliferative diabetic retinopathy without macular edema	1 - Adverse drug events
E09.36	Drug or chemical induced diabetes mellitus with diabetic cataract	1 - Adverse drug events
E09.39	Drug or chemical induced diabetes mellitus with other diabetic ophthalmic complication	1 - Adverse drug events
E09.4	Drug or chemical induced diabetes mellitus with neurological complications	1 - Adverse drug events
E09.40	Drug or chemical induced diabetes mellitus with neurological complications with diabetic neuropathy, unspecified	1 - Adverse drug events
E09.41	Drug or chemical induced diabetes mellitus with neurological complications with diabetic mononeuropathy	1 - Adverse drug events
E09.42	Drug or chemical induced diabetes mellitus with neurological complications with diabetic polyneuropathy	1 - Adverse drug events
E09.43	Drug or chemical induced diabetes mellitus with neurological complications with diabetic autonomic (poly)neuropathy	1 - Adverse drug events
E09.44	Drug or chemical induced diabetes mellitus with neurological complications with diabetic amyotrophy	1 - Adverse drug events
E09.49	Drug or chemical induced diabetes mellitus with neurological complications with other diabetic neurological complication	1 - Adverse drug events
E09.5	Drug or chemical induced diabetes mellitus with circulatory complications	1 - Adverse drug events
E09.51	Drug or chemical induced diabetes mellitus with diabetic peripheral angiopathy without gangrene	1 - Adverse drug events
E09.52	Drug or chemical induced diabetes mellitus with diabetic peripheral angiopathy with gangrene	1 - Adverse drug events
E09.59	Drug or chemical induced diabetes mellitus with other circulatory complications	1 - Adverse drug events
E09.6	Drug or chemical induced diabetes mellitus with other specified complications	1 - Adverse drug events
E09.61	Drug or chemical induced diabetes mellitus with diabetic arthropathy	1 - Adverse drug events
E09.610	Drug or chemical induced diabetes mellitus with diabetic neuropathic arthropathy	1 - Adverse drug events
E09.618	Drug or chemical induced diabetes mellitus with other diabetic arthropathy	1 - Adverse drug events
E09.62	Drug or chemical induced diabetes mellitus with skin complications	1 - Adverse drug events
E09.620	Drug or chemical induced diabetes mellitus with diabetic dermatitis	1 - Adverse drug events
E09.621	Drug or chemical induced diabetes mellitus with foot ulcer	1 - Adverse drug events
E09.622	Drug or chemical induced diabetes mellitus with other skin ulcer	1 - Adverse drug events
E09.628	Drug or chemical induced diabetes mellitus with other skin complications	1 - Adverse drug events
E09.63	Drug or chemical induced diabetes mellitus with oral complications	1 - Adverse drug events
E09.630	Drug or chemical induced diabetes mellitus with periodontal disease	1 - Adverse drug events
E09.638	Drug or chemical induced diabetes mellitus with other oral complications	1 - Adverse drug events
E09.64	Drug or chemical induced diabetes mellitus with hypoglycemia	1 - Adverse drug events
E09.641	Drug or chemical induced diabetes mellitus with hypoglycemia with coma	1 - Adverse drug events
E09.649	Drug or chemical induced diabetes mellitus with hypoglycemia without coma	1 - Adverse drug events
E09.65	Drug or chemical induced diabetes mellitus with hyperglycemia	1 - Adverse drug events
E09.69	Drug or chemical induced diabetes mellitus with other specified complication	1 - Adverse drug events
E09.8	Drug or chemical induced diabetes mellitus with unspecified complications	1 - Adverse drug events
E09.9	Drug or chemical induced diabetes mellitus without complications	1 - Adverse drug events
E16.0	Drug-induced hypoglycemia without coma	1 - Adverse drug events
E23.1	Drug-induced hypopituitarism	1 - Adverse drug events
E24.2	Drug-induced Cushing's syndrome	1 - Adverse drug events
E27.3	Drug-induced adrenocortical insufficiency	1 - Adverse drug events
**E36**	**Intraoperative complications of endocrine system**	2 - Surgical and peri-operative adverse events
**E36**	**Intraoperative complications of endocrine system**	2 - Surgical and peri-operative adverse events
E36.0	Intraoperative hemorrhage and hematoma of an endocrine system organ or structure complicating a procedure	2 - Surgical and peri-operative adverse events
E36.01	Intraoperative hemorrhage and hematoma of an endocrine system organ or structure complicating an endocrine system procedure	2 - Surgical and peri-operative adverse events

© 2019 Sunshine JE et al. *JAMA Network Open*.

E36.02	Intraoperative hemorrhage and hematoma of an endocrine system organ or structure complicating other procedure	2 - Surgical and peri-operative adverse events
E36.1	Accidental puncture and laceration of an endocrine system organ or structure during a procedure	3 - Misadventure
E36.11	Accidental puncture and laceration of an endocrine system organ or structure during an endocrine system procedure	3 - Misadventure
E36.12	Accidental puncture and laceration of an endocrine system organ or structure during other procedure	3 - Misadventure
E36.8	Other intraoperative complications of endocrine system	2 - Surgical and peri-operative adverse events
E66.1	Drug-induced obesity	1 - Adverse drug events
E87.71	Transfusion associated circulatory overload	4 - Medical management
**E89**	**Postprocedural endocrine and metabolic complications and disorders, not elsewhere classified**	2 - Surgical and peri-operative adverse events
E89.0	Postprocedural hypothyroidism	2 - Surgical and peri-operative adverse events
E89.00	Postprocedural endocrine and metabolic complications and disorders, not elsewhere classified	2 - Surgical and peri-operative adverse events
E89.01	Postprocedural endocrine and metabolic complications and disorders, not elsewhere classified	2 - Surgical and peri-operative adverse events
E89.02	Postprocedural endocrine and metabolic complications and disorders, not elsewhere classified	2 - Surgical and peri-operative adverse events
E89.03	Postprocedural endocrine and metabolic complications and disorders, not elsewhere classified	2 - Surgical and peri-operative adverse events
E89.08	Postprocedural endocrine and metabolic complications and disorders, not elsewhere classified	2 - Surgical and peri-operative adverse events
E89.09	Postprocedural endocrine and metabolic complications and disorders, not elsewhere classified	2 - Surgical and peri-operative adverse events
E89.1	Postprocedural hypoinsulinemia	2 - Surgical and peri-operative adverse events
E89.10	Postprocedural endocrine and metabolic complications and disorders, not elsewhere classified	2 - Surgical and peri-operative adverse events
E89.11	Postprocedural endocrine and metabolic complications and disorders, not elsewhere classified	2 - Surgical and peri-operative adverse events
E89.12	Postprocedural endocrine and metabolic complications and disorders, not elsewhere classified	2 - Surgical and peri-operative adverse events
E89.13	Postprocedural endocrine and metabolic complications and disorders, not elsewhere classified	2 - Surgical and peri-operative adverse events
E89.18	Postprocedural endocrine and metabolic complications and disorders, not elsewhere classified	2 - Surgical and peri-operative adverse events
E89.19	Postprocedural endocrine and metabolic complications and disorders, not elsewhere classified	2 - Surgical and peri-operative adverse events
E89.2	Postprocedural hypoparathyroidism	2 - Surgical and peri-operative adverse events
E89.3	Postprocedural hypopituitarism	2 - Surgical and peri-operative adverse events
E89.30	Postprocedural endocrine and metabolic complications and disorders, not elsewhere classified	2 - Surgical and peri-operative adverse events
E89.32	Postprocedural endocrine and metabolic complications and disorders, not elsewhere classified	2 - Surgical and peri-operative adverse events
E89.38	Postprocedural endocrine and metabolic complications and disorders, not elsewhere classified	2 - Surgical and peri-operative adverse events
E89.39	Postprocedural endocrine and metabolic complications and disorders, not elsewhere classified	2 - Surgical and peri-operative adverse events
E89.4	Postprocedural ovarian failure	2 - Surgical and peri-operative adverse events
E89.40	Asymptomatic postprocedural ovarian failure	2 - Surgical and peri-operative adverse events
E89.41	Symptomatic postprocedural ovarian failure	2 - Surgical and peri-operative adverse events
E89.5	Postprocedural testicular hypofunction	2 - Surgical and peri-operative adverse events
E89.6	Postprocedural adrenocortical (-medullary) hypofunction	2 - Surgical and peri-operative adverse events
E89.7	Postprocedural endocrine and metabolic complications and disorders, not elsewhere classified	2 - Surgical and peri-operative adverse events

© 2019 Sunshine JE et al. *JAMA Network Open*.

E89.8	Other postprocedural endocrine and metabolic complications and disorders	2 - Surgical and peri-operative adverse events
E89.80	Postprocedural endocrine and metabolic complications and disorders, not elsewhere classified	2 - Surgical and peri-operative adverse events
E89.81	Postprocedural hemorrhage and hematoma of an endocrine system organ or structure following a procedure	2 - Surgical and peri-operative adverse events
E89.810	Postprocedural hemorrhage and hematoma of an endocrine system organ or structure following an endocrine system procedure	2 - Surgical and peri-operative adverse events
E89.811	Postprocedural hemorrhage and hematoma of an endocrine system organ or structure following other procedure	2 - Surgical and peri-operative adverse events
E89.89	Other postprocedural endocrine and metabolic complications and disorders	2 - Surgical and peri-operative adverse events
E89.9	Postprocedural endocrine and metabolic complications and disorders, not elsewhere classified	2 - Surgical and peri-operative adverse events
G21.1	Other drug-induced secondary parkinsonism	1 - Adverse drug events
G21.11	Neuroleptic induced parkinsonism	1 - Adverse drug events
G21.19	Other drug induced secondary parkinsonism	1 - Adverse drug events
G24.0	Drug induced dystonia	1 - Adverse drug events
G24.01	Drug induced subacute dyskinesia	1 - Adverse drug events
G24.02	Drug induced acute dystonia	1 - Adverse drug events
G24.09	Other drug induced dystonia	1 - Adverse drug events
G25.1	Drug-induced tremor	1 - Adverse drug events
G25.4	Drug-induced chorea	1 - Adverse drug events
G25.6	Drug induced tics and other tics of organic origin	1 - Adverse drug events
G25.61	Drug induced tics	1 - Adverse drug events
G25.69	Other tics of organic origin	6 - Other
G25.7	Other and unspecified drug induced movement disorders	1 - Adverse drug events
G25.70	Drug induced movement disorder, unspecified	1 - Adverse drug events
G25.71	Drug induced akathisia	1 - Adverse drug events
G25.79	Other drug induced movement disorders	1 - Adverse drug events
G72.0	Drug-induced myopathy	1 - Adverse drug events
G93.7	Reye's syndrome	1 - Adverse drug events
**G97**	**Intraoperative and postprocedural complications and disorders of nervous system, not elsewhere classified**	2 - Surgical and peri-operative adverse events
G97.0	Cerebrospinal fluid leak from spinal puncture	2 - Surgical and peri-operative adverse events
G97.1	Other reaction to spinal and lumbar puncture	2 - Surgical and peri-operative adverse events
G97.2	Intracranial hypotension following ventricular shunting	2 - Surgical and peri-operative adverse events
G97.3	Intraoperative hemorrhage and hematoma of a nervous system organ or structure complicating a procedure	2 - Surgical and peri-operative adverse events
G97.31	Intraoperative hemorrhage and hematoma of a nervous system organ or structure complicating a nervous system procedure	2 - Surgical and peri-operative adverse events
G97.32	Intraoperative hemorrhage and hematoma of a nervous system organ or structure complicating other procedure	2 - Surgical and peri-operative adverse events
G97.4	Accidental puncture and laceration of a nervous system organ or structure during a procedure	3 - Misadventure
G97.41	Accidental puncture or laceration of dura during a procedure	3 - Misadventure
G97.48	Accidental puncture and laceration of other nervous system organ or structure during a nervous system procedure	3 - Misadventure
G97.49	Accidental puncture and laceration of other nervous system organ or structure during other procedure	3 - Misadventure
G97.5	Postprocedural hemorrhage and hematoma of a nervous system organ or structure following a procedure	2 - Surgical and peri-operative adverse events
G97.51	Postprocedural hemorrhage and hematoma of a nervous system organ or structure following a nervous system procedure	2 - Surgical and peri-operative adverse events
G97.52	Postprocedural hemorrhage and hematoma of a nervous system organ or structure following other procedure	2 - Surgical and peri-operative adverse events
G97.8	Other intraoperative and postprocedural complications and disorders of nervous system	2 - Surgical and peri-operative adverse events
G97.81	Other intraoperative complications of nervous system	2 - Surgical and peri-operative adverse events
G97.82	Other postprocedural complications and disorders of nervous system	2 - Surgical and peri-operative adverse events

© 2019 Sunshine JE et al. *JAMA Network Open*.

G97.9	Postprocedural disorder of nervous system, unspecified	2 - Surgical and peri-operative adverse events
I95.2	Hypotension due to drugs	1 - Adverse drug events
I95.3	Hypotension of hemodialysis	4 - Medical management
**I97**	**Intraoperative and postprocedural complications and disorders of circulatory system, not elsewhere classified**	2 - Surgical and peri-operative adverse events
I97.0	Postcardiotomy syndrome	2 - Surgical and peri-operative adverse events
I97.1	Other postprocedural cardiac functional disturbances	2 - Surgical and peri-operative adverse events
I97.11	Postprocedural cardiac insufficiency	2 - Surgical and peri-operative adverse events
I97.110	Postprocedural cardiac insufficiency following cardiac surgery	2 - Surgical and peri-operative adverse events
I97.111	Postprocedural cardiac insufficiency following other surgery	2 - Surgical and peri-operative adverse events
I97.12	Postprocedural cardiac arrest	2 - Surgical and peri-operative adverse events
I97.120	Postprocedural cardiac arrest following cardiac surgery	2 - Surgical and peri-operative adverse events
I97.121	Postprocedural cardiac arrest following other surgery	2 - Surgical and peri-operative adverse events
I97.13	Postprocedural heart failure	2 - Surgical and peri-operative adverse events
I97.130	Postprocedural heart failure following cardiac surgery	2 - Surgical and peri-operative adverse events
I97.131	Postprocedural heart failure following other surgery	2 - Surgical and peri-operative adverse events
I97.19	Other postprocedural cardiac functional disturbances	2 - Surgical and peri-operative adverse events
I97.190	Other postprocedural cardiac functional disturbances following cardiac surgery	2 - Surgical and peri-operative adverse events
I97.191	Other postprocedural cardiac functional disturbances following other surgery	2 - Surgical and peri-operative adverse events
I97.2	Postmastectomy lymphedema syndrome	2 - Surgical and peri-operative adverse events
I97.3	Postprocedural hypertension	2 - Surgical and peri-operative adverse events
I97.4	Intraoperative hemorrhage and hematoma of a circulatory system organ or structure complicating a procedure	2 - Surgical and peri-operative adverse events
I97.41	Intraoperative hemorrhage and hematoma of a circulatory system organ or structure complicating a circulatory system procedure	2 - Surgical and peri-operative adverse events
I97.410	Intraoperative hemorrhage and hematoma of a circulatory system organ or structure complicating a cardiac catheterization	2 - Surgical and peri-operative adverse events
I97.411	Intraoperative hemorrhage and hematoma of a circulatory system organ or structure complicating a cardiac bypass	2 - Surgical and peri-operative adverse events
I97.418	Intraoperative hemorrhage and hematoma of a circulatory system organ or structure complicating other circulatory system procedure	2 - Surgical and peri-operative adverse events
I97.42	Intraoperative hemorrhage and hematoma of a circulatory system organ or structure complicating other procedure	2 - Surgical and peri-operative adverse events
I97.5	Accidental puncture and laceration of a circulatory system organ or structure during a procedure	3 - Misadventure
I97.51	Accidental puncture and laceration of a circulatory system organ or structure during a circulatory system procedure	3 - Misadventure
I97.52	Accidental puncture and laceration of a circulatory system organ or structure during other procedure	3 - Misadventure
I97.6	Postprocedural hemorrhage and hematoma of a circulatory system organ or structure following a procedure	2 - Surgical and peri-operative adverse events
I97.61	Postprocedural hemorrhage and hematoma of a circulatory system organ or structure following a circulatory system procedure	2 - Surgical and peri-operative adverse events
I97.610	Postprocedural hemorrhage and hematoma of a circulatory system organ or structure following a cardiac catheterization	2 - Surgical and peri-operative adverse events
I97.611	Postprocedural hemorrhage and hematoma of a circulatory system organ or structure following cardiac bypass	2 - Surgical and peri-operative adverse events
I97.618	Postprocedural hemorrhage and hematoma of a circulatory system organ or structure following other circulatory system procedure	2 - Surgical and peri-operative adverse events

© 2019 Sunshine JE et al. *JAMA Network Open*.

I97.62	Postprocedural hemorrhage and hematoma of a circulatory system organ or structure following other procedure	2 - Surgical and peri-operative adverse events
I97.7	Intraoperative cardiac functional disturbances	2 - Surgical and peri-operative adverse events
I97.71	Intraoperative cardiac arrest	2 - Surgical and peri-operative adverse events
I97.710	Intraoperative cardiac arrest during cardiac surgery	2 - Surgical and peri-operative adverse events
I97.711	Intraoperative cardiac arrest during other surgery	2 - Surgical and peri-operative adverse events
I97.79	Other intraoperative cardiac functional disturbances	2 - Surgical and peri-operative adverse events
I97.790	Other intraoperative cardiac functional disturbances during cardiac surgery	2 - Surgical and peri-operative adverse events
I97.791	Other intraoperative cardiac functional disturbances during other surgery	2 - Surgical and peri-operative adverse events
I97.8	Other intraoperative and postprocedural complications and disorders of the circulatory system, not elsewhere classified	2 - Surgical and peri-operative adverse events
I97.81	Intraoperative cerebrovascular infarction	2 - Surgical and peri-operative adverse events
I97.810	Intraoperative cerebrovascular infarction during cardiac surgery	2 - Surgical and peri-operative adverse events
I97.811	Intraoperative cerebrovascular infarction during other surgery	2 - Surgical and peri-operative adverse events
I97.82	Postprocedural cerebrovascular infarction	2 - Surgical and peri-operative adverse events
I97.820	Postprocedural cerebrovascular infarction during cardiac surgery	2 - Surgical and peri-operative adverse events
I97.821	Postprocedural cerebrovascular infarction during other surgery	2 - Surgical and peri-operative adverse events
I97.88	Other intraoperative complications of the circulatory system, not elsewhere classified	2 - Surgical and peri-operative adverse events
I97.89	Other postprocedural complications and disorders of the circulatory system, not elsewhere classified	2 - Surgical and peri-operative adverse events
I97.9	Postprocedural disorder of circulatory system, unspecified	2 - Surgical and peri-operative adverse events
I98.9		2 - Surgical and peri-operative adverse events
J70.2	Acute drug-induced interstitial lung disorders	1 - Adverse drug events
J70.3	Chronic drug-induced interstitial lung disorders	1 - Adverse drug events
J70.4	Drug-induced interstitial lung disorders, unspecified	1 - Adverse drug events
J70.5	Respiratory conditions due to smoke inhalation	6 - Other
**J95**	**Intraoperative and postprocedural complications and disorders of respiratory system, not elsewhere classified**	2 - Surgical and peri-operative adverse events
J95.0	Tracheostomy complications	2 - Surgical and peri-operative adverse events
J95.00	Unspecified tracheostomy complication	2 - Surgical and peri-operative adverse events
J95.01	Hemorrhage from tracheostomy stoma	2 - Surgical and peri-operative adverse events
J95.02	Infection of tracheostomy stoma	2 - Surgical and peri-operative adverse events
J95.03	Malfunction of tracheostomy stoma	2 - Surgical and peri-operative adverse events
J95.04	Tracheo-esophageal fistula following tracheostomy	2 - Surgical and peri-operative adverse events
J95.09	Other tracheostomy complication	2 - Surgical and peri-operative adverse events
J95.1	Acute pulmonary insufficiency following thoracic surgery	2 - Surgical and peri-operative adverse events
J95.2	Acute pulmonary insufficiency following nonthoracic surgery	2 - Surgical and peri-operative adverse events
J95.3	Chronic pulmonary insufficiency following surgery	2 - Surgical and peri-operative adverse events

© 2019 Sunshine JE et al. *JAMA Network Open*.

J95.4	Chemical pneumonitis due to anesthesia	2 - Surgical and peri-operative adverse events
J95.5	Postprocedural subglottic stenosis	2 - Surgical and peri-operative adverse events
J95.6	Intraoperative hemorrhage and hematoma of a respiratory system organ or structure complicating a procedure	2 - Surgical and peri-operative adverse events
J95.61	Intraoperative hemorrhage and hematoma of a respiratory system organ or structure complicating a respiratory system procedure	2 - Surgical and peri-operative adverse events
J95.62	Intraoperative hemorrhage and hematoma of a respiratory system organ or structure complicating other procedure	2 - Surgical and peri-operative adverse events
J95.7	Accidental puncture and laceration of a respiratory system organ or structure during a procedure	3 - Misadventure
J95.71	Accidental puncture and laceration of a respiratory system organ or structure during a respiratory system procedure	3 - Misadventure
J95.72	Accidental puncture and laceration of a respiratory system organ or structure during other procedure	3 - Misadventure
J95.8	Other intraoperative and postprocedural complications and disorders of respiratory system, not elsewhere classified	2 - Surgical and peri-operative adverse events
J95.81	Postprocedural pneumothorax and air leak	2 - Surgical and peri-operative adverse events
J95.811	Postprocedural pneumothorax	2 - Surgical and peri-operative adverse events
J95.812	Postprocedural air leak	2 - Surgical and peri-operative adverse events
J95.82	Postprocedural respiratory failure	2 - Surgical and peri-operative adverse events
J95.821	Acute postprocedural respiratory failure	2 - Surgical and peri-operative adverse events
J95.822	Acute and chronic postprocedural respiratory failure	2 - Surgical and peri-operative adverse events
J95.83	Postprocedural hemorrhage and hematoma of a respiratory system organ or structure following a procedure	2 - Surgical and peri-operative adverse events
J95.830	Postprocedural hemorrhage and hematoma of a respiratory system organ or structure following a respiratory system procedure	2 - Surgical and peri-operative adverse events
J95.831	Postprocedural hemorrhage and hematoma of a respiratory system organ or structure following other procedure	2 - Surgical and peri-operative adverse events
J95.84	Transfusion-related acute lung injury (TRALI)	4 - Medical management
J95.85	Complication of respirator [ventilator]	4 - Medical management
J95.850	Mechanical complication of respirator	4 - Medical management
J95.851	Ventilator associated pneumonia	4 - Medical management
J95.859	Other complication of respirator [ventilator]	4 - Medical management
J95.88	Other intraoperative complications of respiratory system, not elsewhere classified	2 - Surgical and peri-operative adverse events
J95.89	Other postprocedural complications and disorders of respiratory system, not elsewhere classified	2 - Surgical and peri-operative adverse events
J95.9	Postprocedural respiratory disorder, unspecified	2 - Surgical and peri-operative adverse events
**K43**	**Ventral hernia**	2 - Surgical and peri-operative adverse events
K43.0	Incisional hernia with obstruction, without gangrene	2 - Surgical and peri-operative adverse events
K43.1	Incisional hernia with gangrene	2 - Surgical and peri-operative adverse events
K43.2	Incisional hernia without obstruction or gangrene	2 - Surgical and peri-operative adverse events
K43.3	Parastomal hernia with obstruction, without gangrene	2 - Surgical and peri-operative adverse events
K43.4	Parastomal hernia with gangrene	2 - Surgical and peri-operative adverse events
K43.5	Parastomal hernia without obstruction or gangrene	2 - Surgical and peri-operative adverse events
K43.6	Other and unspecified ventral hernia with obstruction, without gangrene	2 - Surgical and peri-operative adverse events
K43.7	Other and unspecified ventral hernia with gangrene	2 - Surgical and peri-operative adverse events

© 2019 Sunshine JE et al. *JAMA Network Open*.

K43.9	Ventral hernia without obstruction or gangrene	2 - Surgical and peri-operative adverse events
K62.7	Radiation proctitis	4 - Medical management
**K91**	**Intraoperative and postprocedural complications and disorders of digestive system, not elsewhere classified**	2 - Surgical and peri-operative adverse events
K91.0	Vomiting following gastrointestinal surgery	2 - Surgical and peri-operative adverse events
K91.1	Postgastric surgery syndromes	2 - Surgical and peri-operative adverse events
K91.2	Postsurgical malabsorption, not elsewhere classified	2 - Surgical and peri-operative adverse events
K91.3	Postprocedural intestinal obstruction	2 - Surgical and peri-operative adverse events
K91.4	Colostomy and enterostomy malfunction	2 - Surgical and peri-operative adverse events
K91.5	Postcholecystectomy syndrome	2 - Surgical and peri-operative adverse events
K91.6	Intraoperative hemorrhage and hematoma of a digestive system organ or structure complicating a procedure	2 - Surgical and peri-operative adverse events
K91.61	Intraoperative hemorrhage and hematoma of a digestive system organ or structure complicating a digestive sytem procedure	2 - Surgical and peri-operative adverse events
K91.62	Intraoperative hemorrhage and hematoma of a digestive system organ or structure complicating other procedure	2 - Surgical and peri-operative adverse events
K91.7	Accidental puncture and laceration of a digestive system organ or structure during a procedure	3 - Misadventure
K91.71	Accidental puncture and laceration of a digestive system organ or structure during a digestive system procedure	3 - Misadventure
K91.72	Accidental puncture and laceration of a digestive system organ or structure during other procedure	3 - Misadventure
K91.8	Other intraoperative and postprocedural complications and disorders of digestive system	2 - Surgical and peri-operative adverse events
K91.81	Other intraoperative complications of digestive system	2 - Surgical and peri-operative adverse events
K91.82	Postprocedural hepatic failure	2 - Surgical and peri-operative adverse events
K91.83	Postprocedural hepatorenal syndrome	2 - Surgical and peri-operative adverse events
K91.84	Postprocedural hemorrhage and hematoma of a digestive system organ or structure following a procedure	2 - Surgical and peri-operative adverse events
K91.840	Postprocedural hemorrhage and hematoma of a digestive system organ or structure following a digestive system procedure	2 - Surgical and peri-operative adverse events
K91.841	Postprocedural hemorrhage and hematoma of a digestive system organ or structure following other procedure	2 - Surgical and peri-operative adverse events
K91.85	Complications of intestinal pouch	2 - Surgical and peri-operative adverse events
K91.850	Pouchitis	2 - Surgical and peri-operative adverse events
K91.858	Other complications of intestinal pouch	2 - Surgical and peri-operative adverse events
K91.86	Retained cholelithiasis following cholecystectomy	2 - Surgical and peri-operative adverse events
K91.89	Other postprocedural complications and disorders of digestive system	2 - Surgical and peri-operative adverse events
K91.9	Postprocedural disorder of digestive system, unspecified	2 - Surgical and peri-operative adverse events
**K94**	**Complications of artificial openings of the digestive system**	2 - Surgical and peri-operative adverse events
K94.0	Colostomy complications	2 - Surgical and peri-operative adverse events
K94.00	Colostomy complication, unspecified	2 - Surgical and peri-operative adverse events
K94.01	Colostomy hemorrhage	2 - Surgical and peri-operative adverse events
K94.02	Colostomy infection	2 - Surgical and peri-operative adverse events

© 2019 Sunshine JE et al. *JAMA Network Open*.

K94.03	Colostomy malfunction	2 - Surgical and peri-operative adverse events
K94.09	Other complications of colostomy	2 - Surgical and peri-operative adverse events
K94.1	Enterostomy complications	2 - Surgical and peri-operative adverse events
K94.10	Enterostomy complication, unspecified	2 - Surgical and peri-operative adverse events
K94.11	Enterostomy hemorrhage	2 - Surgical and peri-operative adverse events
K94.12	Enterostomy infection	2 - Surgical and peri-operative adverse events
K94.13	Enterostomy malfunction	2 - Surgical and peri-operative adverse events
K94.19	Other complications of enterostomy	2 - Surgical and peri-operative adverse events
K94.2	Gastrostomy complications	2 - Surgical and peri-operative adverse events
K94.20	Gastrostomy complication, unspecified	2 - Surgical and peri-operative adverse events
K94.21	Gastrostomy hemorrhage	2 - Surgical and peri-operative adverse events
K94.22	Gastrostomy infection	2 - Surgical and peri-operative adverse events
K94.23	Gastrostomy malfunction	2 - Surgical and peri-operative adverse events
K94.29	Other complications of gastrostomy	2 - Surgical and peri-operative adverse events
K94.3	Esophagostomy complications	2 - Surgical and peri-operative adverse events
K94.30	Esophagostomy complications, unspecified	2 - Surgical and peri-operative adverse events
K94.31	Esophagostomy hemorrhage	2 - Surgical and peri-operative adverse events
K94.32	Esophagostomy infection	2 - Surgical and peri-operative adverse events
K94.33	Esophagostomy malfunction	2 - Surgical and peri-operative adverse events
K94.39	Other complications of esophagostomy	2 - Surgical and peri-operative adverse events
**K95**	**Complications of bariatric procedures**	2 - Surgical and peri-operative adverse events
K95.0	Complications of gastric band procedure	2 - Surgical and peri-operative adverse events
K95.01	Infection due to gastric band procedure	2 - Surgical and peri-operative adverse events
K95.09	Other complications of gastric band procedure	2 - Surgical and peri-operative adverse events
K95.8	Complications of other bariatric procedure	2 - Surgical and peri-operative adverse events
K95.81	Infection due to other bariatric procedure	2 - Surgical and peri-operative adverse events
K95.89	Other complications of other bariatric procedure	2 - Surgical and peri-operative adverse events
M87.1	Osteonecrosis due to drugs	1 - Adverse drug events
M87.10	Osteonecrosis due to drugs, unspecified bone	1 - Adverse drug events
M87.11	Osteonecrosis due to drugs, shoulder	1 - Adverse drug events
M87.111	Osteonecrosis due to drugs, right shoulder	1 - Adverse drug events
M87.112	Osteonecrosis due to drugs, left shoulder	1 - Adverse drug events
M87.119	Osteonecrosis due to drugs, unspecified shoulder	1 - Adverse drug events
M87.12	Osteonecrosis due to drugs, humerus	1 - Adverse drug events
M87.121	Osteonecrosis due to drugs, right humerus	1 - Adverse drug events
M87.122	Osteonecrosis due to drugs, left humerus	1 - Adverse drug events
M87.129	Osteonecrosis due to drugs, unspecified humerus	1 - Adverse drug events

© 2019 Sunshine JE et al. *JAMA Network Open*.

M87.13	Osteonecrosis due to drugs of radius, ulna and carpus	1 - Adverse drug events
M87.131	Osteonecrosis due to drugs of right radius	1 - Adverse drug events
M87.132	Osteonecrosis due to drugs of left radius	1 - Adverse drug events
M87.133	Osteonecrosis due to drugs of unspecified radius	1 - Adverse drug events
M87.134	Osteonecrosis due to drugs of right ulna	1 - Adverse drug events
M87.135	Osteonecrosis due to drugs of left ulna	1 - Adverse drug events
M87.136	Osteonecrosis due to drugs of unspecified ulna	1 - Adverse drug events
M87.137	Osteonecrosis due to drugs of right carpus	1 - Adverse drug events
M87.138	Osteonecrosis due to drugs of left carpus	1 - Adverse drug events
M87.139	Osteonecrosis due to drugs of unspecified carpus	1 - Adverse drug events
M87.14	Osteonecrosis due to drugs, hand and fingers	1 - Adverse drug events
M87.141	Osteonecrosis due to drugs, right hand	1 - Adverse drug events
M87.142	Osteonecrosis due to drugs, left hand	1 - Adverse drug events
M87.143	Osteonecrosis due to drugs, unspecified hand	1 - Adverse drug events
M87.144	Osteonecrosis due to drugs, right finger(s)	1 - Adverse drug events
M87.145	Osteonecrosis due to drugs, left finger(s)	1 - Adverse drug events
M87.146	Osteonecrosis due to drugs, unspecified finger(s)	1 - Adverse drug events
M87.15	Osteonecrosis due to drugs, pelvis and femur	1 - Adverse drug events
M87.150	Osteonecrosis due to drugs, pelvis	1 - Adverse drug events
M87.151	Osteonecrosis due to drugs, right femur	1 - Adverse drug events
M87.152	Osteonecrosis due to drugs, left femur	1 - Adverse drug events
M87.159	Osteonecrosis due to drugs, unspecified femur	1 - Adverse drug events
M87.16	Osteonecrosis due to drugs, tibia and fibula	1 - Adverse drug events
M87.161	Osteonecrosis due to drugs, right tibia	1 - Adverse drug events
M87.162	Osteonecrosis due to drugs, left tibia	1 - Adverse drug events
M87.163	Osteonecrosis due to drugs, unspecified tibia	1 - Adverse drug events
M87.164	Osteonecrosis due to drugs, right fibula	1 - Adverse drug events
M87.165	Osteonecrosis due to drugs, left fibula	1 - Adverse drug events
M87.166	Osteonecrosis due to drugs, unspecified fibula	1 - Adverse drug events
M87.17	Osteonecrosis due to drugs, ankle, foot and toes	1 - Adverse drug events
M87.171	Osteonecrosis due to drugs, right ankle	1 - Adverse drug events
M87.172	Osteonecrosis due to drugs, left ankle	1 - Adverse drug events
M87.173	Osteonecrosis due to drugs, unspecified ankle	1 - Adverse drug events
M87.174	Osteonecrosis due to drugs, right foot	1 - Adverse drug events
M87.175	Osteonecrosis due to drugs, left foot	1 - Adverse drug events
M87.176	Osteonecrosis due to drugs, unspecified foot	1 - Adverse drug events
M87.177	Osteonecrosis due to drugs, right toe(s)	1 - Adverse drug events
M87.178	Osteonecrosis due to drugs, left toe(s)	1 - Adverse drug events
M87.179	Osteonecrosis due to drugs, unspecified toe(s)	1 - Adverse drug events
M87.18	Osteonecrosis due to drugs, other site	1 - Adverse drug events
M87.180	Osteonecrosis due to drugs, jaw	1 - Adverse drug events
M87.188	Osteonecrosis due to drugs, other site	1 - Adverse drug events
M87.19	Osteonecrosis due to drugs, multiple sites	1 - Adverse drug events
**N14**	**Drug- and heavy-metal-induced tubulo-interstitial and tubular conditions**	1 - Adverse drug events
N14.0	Analgesic nephropathy	1 - Adverse drug events
N14.1	Nephropathy induced by other drugs, medicaments and biological substances	1 - Adverse drug events
N14.2	Nephropathy induced by unspecified drug, medicament or biological substance	1 - Adverse drug events
N14.3	Nephropathy induced by heavy metals	6 - Other
N14.4	Toxic nephropathy, not elsewhere classified	6 - Other
**N65**	**Deformity and disproportion of reconstructed breast**	2 - Surgical and peri-operative adverse events
N65.0	Deformity of reconstructed breast	2 - Surgical and peri-operative adverse events
N65.1	Disproportion of reconstructed breast	2 - Surgical and peri-operative adverse events
**N99**	**Intraoperative and postprocedural complications and disorders of genitourinary system, not elsewhere classified**	2 - Surgical and peri-operative adverse events
N99.0	Postprocedural (acute) (chronic) kidney failure	2 - Surgical and peri-operative adverse events
N99.1	Postprocedural urethral stricture	2 - Surgical and peri-operative adverse events

© 2019 Sunshine JE et al. *JAMA Network Open*.

N99.11	Postprocedural urethral stricture, male	2 - Surgical and peri-operative adverse events
N99.110	Postprocedural urethral stricture, male, meatal	2 - Surgical and peri-operative adverse events
N99.111	Postprocedural bulbous urethral stricture	2 - Surgical and peri-operative adverse events
N99.112	Postprocedural membranous urethral stricture	2 - Surgical and peri-operative adverse events
N99.113	Postprocedural anterior urethral stricture	2 - Surgical and peri-operative adverse events
N99.114	Postprocedural urethral stricture, male, unspecified	2 - Surgical and peri-operative adverse events
N99.12	Postprocedural urethral stricture, female	2 - Surgical and peri-operative adverse events
N99.2	Postprocedural adhesions of vagina	2 - Surgical and peri-operative adverse events
N99.3	Prolapse of vaginal vault after hysterectomy	2 - Surgical and peri-operative adverse events
N99.4	Postprocedural pelvic peritoneal adhesions	2 - Surgical and peri-operative adverse events
N99.5	Complications of stoma of urinary tract	2 - Surgical and peri-operative adverse events
N99.51	Complication of cystostomy	2 - Surgical and peri-operative adverse events
N99.510	Cystostomy hemorrhage	2 - Surgical and peri-operative adverse events
N99.511	Cystostomy infection	2 - Surgical and peri-operative adverse events
N99.512	Cystostomy malfunction	2 - Surgical and peri-operative adverse events
N99.518	Other cystostomy complication	2 - Surgical and peri-operative adverse events
N99.52	Complication of other external stoma of urinary tract	2 - Surgical and peri-operative adverse events
N99.520	Hemorrhage of other external stoma of urinary tract	2 - Surgical and peri-operative adverse events
N99.521	Infection of other external stoma of urinary tract	2 - Surgical and peri-operative adverse events
N99.522	Malfunction of other external stoma of urinary tract	2 - Surgical and peri-operative adverse events
N99.528	Other complication of other external stoma of urinary tract	2 - Surgical and peri-operative adverse events
N99.53	Complication of other stoma of urinary tract	2 - Surgical and peri-operative adverse events
N99.530	Hemorrhage of other stoma of urinary tract	2 - Surgical and peri-operative adverse events
N99.531	Infection of other stoma of urinary tract	2 - Surgical and peri-operative adverse events
N99.532	Malfunction of other stoma of urinary tract	2 - Surgical and peri-operative adverse events
N99.538	Other complication of other stoma of urinary tract	2 - Surgical and peri-operative adverse events
N99.6	Intraoperative hemorrhage and hematoma of a genitourinary system organ or structure complicating a procedure	2 - Surgical and peri-operative adverse events
N99.61	Intraoperative hemorrhage and hematoma of a genitourinary system organ or structure complicating a genitourinary system procedure	2 - Surgical and peri-operative adverse events
N99.62	Intraoperative hemorrhage and hematoma of a genitourinary system organ or structure complicating other procedure	2 - Surgical and peri-operative adverse events
N99.7	Accidental puncture and laceration of a genitourinary system organ or structure during a procedure	3 - Misadventure
N99.71	Accidental puncture and laceration of a genitourinary system organ or structure during a genitourinary system procedure	3 - Misadventure
N99.72	Accidental puncture and laceration of a genitourinary system organ or structure during other procedure	3 - Misadventure
N99.8	Other intraoperative and postprocedural complications and disorders of genitourinary system	2 - Surgical and peri-operative adverse events

© 2019 Sunshine JE et al. *JAMA Network Open*.

N99.81	Other intraoperative complications of genitourinary system	2 - Surgical and peri-operative adverse events
N99.82	Postprocedural hemorrhage and hematoma of a genitourinary system organ or structure following a procedure	2 - Surgical and peri-operative adverse events
N99.820	Postprocedural hemorrhage and hematoma of a genitourinary system organ or structure following a genitourinary system procedure	2 - Surgical and peri-operative adverse events
N99.821	Postprocedural hemorrhage and hematoma of a genitourinary system organ or structure following other procedure	2 - Surgical and peri-operative adverse events
N99.83	Residual ovary syndrome	2 - Surgical and peri-operative adverse events
N99.89	Other postprocedural complications and disorders of genitourinary system	2 - Surgical and peri-operative adverse events
N99.9		2 - Surgical and peri-operative adverse events
P96.2	Withdrawal symptoms from therapeutic use of drugs in newborn	1 - Adverse drug events
P96.5	Complication to newborn due to (fetal) intrauterine procedure	2 - Surgical and peri-operative adverse events
R50.2	Drug induced fever	1 - Adverse drug events
R50.82	Postprocedural fever	2 - Surgical and peri-operative adverse events
R50.83	Postvaccination fever	1 - Adverse drug events
**Y40**	**Systemic antibiotics**	1 - Adverse drug events
Y40.0	Penicillins	1 - Adverse drug events
Y40.1	Cefalosporins and other beta-lactam antibiotics	1 - Adverse drug events
Y40.2	Chloramphenicol group	1 - Adverse drug events
Y40.3	Macrolides	1 - Adverse drug events
Y40.4	Tetracyclines	1 - Adverse drug events
Y40.5	Aminoglycosides	1 - Adverse drug events
Y40.6	Rifamycins	1 - Adverse drug events
Y40.7	Antifungal antibiotics, systemically used	1 - Adverse drug events
Y40.8	Other systemic antibiotics	1 - Adverse drug events
Y40.9	Systemic antibiotic, unspecified	1 - Adverse drug events
**Y41**	**Other systemic anti-infectives and antiparasitics**	1 - Adverse drug events
Y41.0	Sulfonamides	1 - Adverse drug events
Y41.1	Antimycobacterial drugs	1 - Adverse drug events
Y41.2	Antimalarials and drugs acting on other blood protozoa	1 - Adverse drug events
Y41.3	Other antiprotozoal drugs	1 - Adverse drug events
Y41.4	Anthelminthics	1 - Adverse drug events
Y41.5	Antiviral drugs	1 - Adverse drug events
Y41.8	Other specified systemic anti-infectives and antiparasitics	1 - Adverse drug events
Y41.9	Systemic anti-infective and antiparasitic, unspecified	1 - Adverse drug events
**Y42**	**Hormones and their synthetic substitutes and antagonists, not elsewhere classified**	1 - Adverse drug events
Y42.0	Glucocorticoids and synthetic analogues	1 - Adverse drug events
Y42.1	Thyroid hormones and substitutes	1 - Adverse drug events
Y42.2	Antithyroid drugs	1 - Adverse drug events
Y42.3	Insulin and oral hypoglycaemic [antidiabetic] drugs	1 - Adverse drug events
Y42.4	Oral contraceptives	1 - Adverse drug events
Y42.5	Other estrogens and progestogens	1 - Adverse drug events
Y42.6	Antigonadotrophins, antiestrogens, antiandrogens, not elsewhere classified	1 - Adverse drug events
Y42.7	Androgens and anabolic congeners	1 - Adverse drug events
Y42.8	Other and unspecified hormones and their synthetic substitutes	1 - Adverse drug events
Y42.9	Other and unspecified hormone antagonists	1 - Adverse drug events
**Y43**	**Primarily systemic agents**	1 - Adverse drug events
Y43.0	Antiallergic and antiemetic drugs	1 - Adverse drug events
Y43.1	Antineoplastic antimetabolites	1 - Adverse drug events
Y43.2	Antineoplastic natural products	1 - Adverse drug events
Y43.3	Other antineoplastic drugs	1 - Adverse drug events
Y43.4	Immunosuppressive agents	1 - Adverse drug events
Y43.5	Acidifying and alkalizing agents	1 - Adverse drug events
Y43.6	Enzymes, not elsewhere classified	1 - Adverse drug events
Y43.8	Other primarily systemic agents, not elsewhere classified	1 - Adverse drug events
Y43.9	Primarily systemic agent, unspecified	1 - Adverse drug events

© 2019 Sunshine JE et al. *JAMA Network Open*.

**Y44**	**Agents primarily affecting blood constituents**	1 - Adverse drug events
Y44.0	Iron preparations and other anti-hypochromic-anaemia preparations	1 - Adverse drug events
Y44.1	Vitamin B 12 , folic acid and other anti-megaloblastic-anaemia preparations	1 - Adverse drug events
Y44.2	Anticoagulants	1 - Adverse drug events
Y44.3	Anticoagulant antagonists, vitamin K and other coagulants	1 - Adverse drug events
Y44.4	Antithrombotic drugs [platelet-aggregation inhibitors]	1 - Adverse drug events
Y44.5	Thrombolytic drugs	1 - Adverse drug events
Y44.6	Natural blood and blood products	1 - Adverse drug events
Y44.7	Plasma substitutes	1 - Adverse drug events
Y44.9	Other and unspecified agents affecting blood constituents	1 - Adverse drug events
**Y45**	**Analgesics, antipyretics and anti-inflammatory drugs**	1 - Adverse drug events
Y45.0	Opioids and related analgesics	1 - Adverse drug events
Y45.1	Salicylates	1 - Adverse drug events
Y45.2	Propionic acid derivatives	1 - Adverse drug events
Y45.3	Other nonsteroidal anti-inflammatory drugs [NSAID]	1 - Adverse drug events
Y45.4	Antirheumatics	1 - Adverse drug events
Y45.5	4-Aminophenol derivatives	1 - Adverse drug events
Y45.8	Other analgesics and antipyretics	1 - Adverse drug events
Y45.9	Analgesic, antipyretic and anti-inflammatory drug, unspecified	1 - Adverse drug events
**Y46**	**Antiepileptics and antiparkinsonism drugs**	1 - Adverse drug events
Y46.0	Succinimides	1 - Adverse drug events
Y46.1	Oxazolidinediones	1 - Adverse drug events
Y46.2	Hydantoin derivatives	1 - Adverse drug events
Y46.3	Deoxybarbiturates	1 - Adverse drug events
Y46.4	Iminostilbenes	1 - Adverse drug events
Y46.5	Valproic acid	1 - Adverse drug events
Y46.6	Other and unspecified antiepileptics	1 - Adverse drug events
Y46.7	Antiparkinsonism drugs	1 - Adverse drug events
Y46.8	Antispasticity drugs	1 - Adverse drug events
**Y47**	**Sedatives, hypnotics and antianxiety drugs**	1 - Adverse drug events
Y47.0	Barbiturates, not elsewhere classified	1 - Adverse drug events
Y47.1	Benzodiazepines	1 - Adverse drug events
Y47.2	Cloral derivatives	1 - Adverse drug events
Y47.3	Paraldehyde	1 - Adverse drug events
Y47.4	Bromine compounds	1 - Adverse drug events
Y47.5	Mixed sedatives and hypnotics, not elsewhere classified	1 - Adverse drug events
Y47.8	Other sedatives, hypnotics and antianxiety drugs	1 - Adverse drug events
Y47.9	Sedative, hypnotic and antianxiety drug, unspecified	1 - Adverse drug events
**Y48**	**Anaesthetics and therapeutic gases**	1 - Adverse drug events
Y48.0	Inhaled anaesthetics	1 - Adverse drug events
Y48.1	Parenteral anaesthetics	1 - Adverse drug events
Y48.2	Other and unspecified general anaesthetics	1 - Adverse drug events
Y48.3	Local anaesthetics	1 - Adverse drug events
Y48.4	Anaesthetic, unspecified	1 - Adverse drug events
Y48.5	Therapeutic gases	1 - Adverse drug events
**Y49**	**Psychotropic drugs, not elsewhere classified**	1 - Adverse drug events
Y49.0	Tricyclic and tetracyclic antidepressants	1 - Adverse drug events
Y49.1	Monoamine-oxidase-inhibitor antidepressants	1 - Adverse drug events
Y49.2	Other and unspecified antidepressants	1 - Adverse drug events
Y49.3	Phenothiazine antipsychotics and neuroleptics	1 - Adverse drug events
Y49.4	Butyrophenone and thioxanthene neuroleptics	1 - Adverse drug events
Y49.5	Other antipsychotics and neuroleptics	1 - Adverse drug events
Y49.6	Psychodysleptics [hallucinogens]	1 - Adverse drug events
Y49.7	Psychostimulants with abuse potential	1 - Adverse drug events
Y49.8	Other psychotropic drugs, not elsewhere classified	1 - Adverse drug events
Y49.9	Psychotropic drug, unspecified	1 - Adverse drug events
**Y50**	**Central nervous system stimulants, not elsewhere classified**	1 - Adverse drug events
Y50.0	Analeptics	1 - Adverse drug events
Y50.1	Opioid receptor antagonists	1 - Adverse drug events
Y50.2	Methylxanthines, not elsewhere classified	1 - Adverse drug events
Y50.8	Other central nervous system stimulants	1 - Adverse drug events

© 2019 Sunshine JE et al. *JAMA Network Open*.

Y50.9	Central nervous system stimulant, unspecified	1 - Adverse drug events
**Y51**	**Drugs primarily affecting the autonomic nervous system**	1 - Adverse drug events
Y51.0	Anticholinesterase agents	1 - Adverse drug events
Y51.1	Other parasympathomimetics [cholinergics]	1 - Adverse drug events
Y51.2	Ganglionic blocking drugs, not elsewhere classified	1 - Adverse drug events
Y51.3	Other parasympatholytics [anticholinergics and antimuscarinics] and spasmolytics, not elsewhere classified	1 - Adverse drug events
Y51.4	Predominantly alpha-adrenoreceptor agonists, not elsewhere classified	1 - Adverse drug events
Y51.5	Predominantly beta-adrenoreceptor agonists, not elsewhere classified	1 - Adverse drug events
Y51.6	Alpha-adrenoreceptor antagonists, not elsewhere classified	1 - Adverse drug events
Y51.7	Beta-adrenoreceptor antagonists, not elsewhere classified	1 - Adverse drug events
Y51.8	Centrally acting and adrenergic-neuron-blocking agents, not elsewhere classified	1 - Adverse drug events
Y51.9	Other and unspecified drugs primarily affecting the autonomic nervous system	1 - Adverse drug events
**Y52**	**Agents primarily affecting the cardiovascular system**	1 - Adverse drug events
Y52.0	Cardiac-stimulant glycosides and drugs of similar action	1 - Adverse drug events
Y52.1	Calcium-channel blockers	1 - Adverse drug events
Y52.2	Other antidysrhythmic drugs, not elsewhere classified	1 - Adverse drug events
Y52.3	Coronary vasodilators, not elsewhere classified	1 - Adverse drug events
Y52.4	Angiotensin-converting-enzyme inhibitors	1 - Adverse drug events
Y52.5	Other antihypertensive drugs, not elsewhere classified	1 - Adverse drug events
Y52.6	Antihyperlipidaemic and antiarteriosclerotic drugs	1 - Adverse drug events
Y52.7	Peripheral vasodilators	1 - Adverse drug events
Y52.8	Antivaricose drugs, including sclerosing agents	1 - Adverse drug events
Y52.9	Other and unspecified agents primarily affecting the cardiovascular system	1 - Adverse drug events
**Y53**	**Agents primarily affecting the gastrointestinal system**	1 - Adverse drug events
Y53.0	Histamine H 2 -receptor antagonists	1 - Adverse drug events
Y53.1	Other antacids and anti-gastric-secretion drugs	1 - Adverse drug events
Y53.2	Stimulant laxatives	1 - Adverse drug events
Y53.3	Saline and osmotic laxatives	1 - Adverse drug events
Y53.4	Other laxatives	1 - Adverse drug events
Y53.5	Digestants	1 - Adverse drug events
Y53.6	Antidiarrhoeal drugs	1 - Adverse drug events
Y53.7	Emetics	1 - Adverse drug events
Y53.8	Other agents primarily affecting the gastrointestinal system	1 - Adverse drug events
Y53.9	Agent primarily affecting the gastrointestinal system, unspecified	1 - Adverse drug events
**Y54**	**Agents primarily affecting water-balance and mineral and uric acid metabolism**	1 - Adverse drug events
Y54.0	Mineralocorticoids	1 - Adverse drug events
Y54.1	Mineralocorticoid antagonists [aldosterone antagonists]	1 - Adverse drug events
Y54.2	Carbonic-anhydrase inhibitors	1 - Adverse drug events
Y54.3	Benzothiadiazine derivatives	1 - Adverse drug events
Y54.4	Loop [high-ceiling] diuretics	1 - Adverse drug events
Y54.5	Other diuretics	1 - Adverse drug events
Y54.6	Electrolytic, caloric and water-balance agents	1 - Adverse drug events
Y54.7	Agents affecting calcification	1 - Adverse drug events
Y54.8	Agents affecting uric acid metabolism	1 - Adverse drug events
Y54.9	Mineral salts, not elsewhere classified	1 - Adverse drug events
**Y55**	**Agents primarily acting on smooth and skeletal muscles and the respiratory system**	1 - Adverse drug events
Y55.0	Oxytocic drugs	1 - Adverse drug events
Y55.1	Skeletal muscle relaxants [neuromuscular blocking agents]	1 - Adverse drug events
Y55.2	Other and unspecified agents primarily acting on muscles	1 - Adverse drug events
Y55.3	Antitussives	1 - Adverse drug events
Y55.4	Expectorants	1 - Adverse drug events
Y55.5	Anti-common-cold drugs	1 - Adverse drug events
Y55.6	Antiasthmatics, not elsewhere classified	1 - Adverse drug events
Y55.7	Other and unspecified agents primarily acting on the respiratory system	1 - Adverse drug events
**Y56**	**Topical agents primarily affecting skin and mucous membrane and ophthalmological, otorhinolaryngological and dental drugs**	1 - Adverse drug events
Y56.0	Local antifungal, anti-infective and anti-inflammatory drugs, not elsewhere classified	1 - Adverse drug events
Y56.1	Antipruritics	1 - Adverse drug events
Y56.2	Local astringents and local detergents	1 - Adverse drug events
Y56.3	Emollients, demulcents and protectants	1 - Adverse drug events

© 2019 Sunshine JE et al. *JAMA Network Open*.

Y56.4	Keratolytics, keratoplastics and other hair treatment drugs and preparations	1 - Adverse drug events
Y56.5	Ophthalmological drugs and preparations	1 - Adverse drug events
Y56.6	Otorhinolaryngological drugs and preparations	1 - Adverse drug events
Y56.7	Dental drugs, topically applied	1 - Adverse drug events
Y56.8	Other topical agents	1 - Adverse drug events
Y56.9	Topical agent, unspecified	1 - Adverse drug events
**Y57**	**Other and unspecified drugs and medicaments**	1 - Adverse drug events
Y57.0	Appetite depressants [anorectics]	1 - Adverse drug events
Y57.1	Lipotropic drugs	1 - Adverse drug events
Y57.2	Antidotes and chelating agents, not elsewhere classified	1 - Adverse drug events
Y57.3	Alcohol deterrents	1 - Adverse drug events
Y57.4	Pharmaceutical excipients	1 - Adverse drug events
Y57.5	X-ray contrast media	1 - Adverse drug events
Y57.6	Other diagnostic agents	1 - Adverse drug events
Y57.7	Vitamins, not elsewhere classified	1 - Adverse drug events
Y57.8	Other drugs and medicaments	1 - Adverse drug events
Y57.9	Drug or medicament, unspecified	1 - Adverse drug events
**Y58**	**Bacterial vaccines**	1 - Adverse drug events
Y58.0	BCG vaccine	1 - Adverse drug events
Y58.1	Typhoid and paratyphoid vaccine	1 - Adverse drug events
Y58.2	Cholera vaccine	1 - Adverse drug events
Y58.3	Plague vaccine	1 - Adverse drug events
Y58.4	Tetanus vaccine	1 - Adverse drug events
Y58.5	Diphtheria vaccine	1 - Adverse drug events
Y58.6	Pertussis vaccine, including combinations with a pertussis component	1 - Adverse drug events
Y58.8	Mixed bacterial vaccines, except combinations with a pertussis component	1 - Adverse drug events
Y58.9	Other and unspecified bacterial vaccines	1 - Adverse drug events
**Y59**	**Other and unspecified vaccines and biological substances**	1 - Adverse drug events
Y59.0	Viral vaccines	1 - Adverse drug events
Y59.1	Rickettsial vaccines	1 - Adverse drug events
Y59.2	Protozoal vaccines	1 - Adverse drug events
Y59.3	Immunoglobulin	1 - Adverse drug events
Y59.8	Other specified vaccines and biological substances	1 - Adverse drug events
Y59.9	Vaccine or biological substance, unspecified	1 - Adverse drug events
**Y60**	**Unintentional cut, puncture, perforation or haemorrhage during surgical and medical care**	3 - Misadventure
Y60.0	During surgical operation	3 - Misadventure
Y60.1	During infusion or transfusion	3 - Misadventure
Y60.2	During kidney dialysis or other perfusion	3 - Misadventure
Y60.3	During injection or immunization	3 - Misadventure
Y60.4	During endoscopic examination	3 - Misadventure
Y60.5	During heart catheterization	3 - Misadventure
Y60.6	During aspiration, puncture and other catheterization	3 - Misadventure
Y60.7	During administration of enema	3 - Misadventure
Y60.8	During other surgical and medical care	3 - Misadventure
Y60.9	During unspecified surgical and medical care	3 - Misadventure
**Y61**	**Foreign object accidentally left in body during surgical and medical care**	3 - Misadventure
Y61.0	During surgical operation	3 - Misadventure
Y61.1	During infusion or transfusion	3 - Misadventure
Y61.2	During kidney dialysis or other perfusion	3 - Misadventure
Y61.3	During injection or immunization	3 - Misadventure
Y61.4	During endoscopic examination	3 - Misadventure
Y61.5	During heart catheterization	3 - Misadventure
Y61.6	During aspiration, puncture and other catheterization	3 - Misadventure
Y61.7	During removal of catheter or packing	3 - Misadventure
Y61.8	During other surgical and medical care	3 - Misadventure
Y61.9	During unspecified surgical and medical care	3 - Misadventure
**Y62**	**Failure of sterile precautions during surgical and medical care**	3 - Misadventure
Y62.0	Failure of sterile precautions during surgical operation	3 - Misadventure
Y62.1	Failure of sterile precautions during infusion or transfusion	3 - Misadventure
Y62.2	Failure of sterile precautions during kidney dialysis and other perfusion	3 - Misadventure
Y62.3	Failure of sterile precautions during injection or immunization	3 - Misadventure

© 2019 Sunshine JE et al. *JAMA Network Open*.

Y62.4	Failure of sterile precautions during endoscopic examination	3 - Misadventure
Y62.5	Failure of sterile precautions during heart catheterization	3 - Misadventure
Y62.6	Failure of sterile precautions during aspiration, puncture and other catheterization	3 - Misadventure
Y62.8	Failure of sterile precautions during other surgical and medical care	3 - Misadventure
Y62.9	Failure of sterile precautions during unspecified surgical and medical care	3 - Misadventure
**Y63**	**Failure in dosage during surgical and medical care**	3 - Misadventure
Y63.0	Excessive amount of blood or other fluid given during transfusion or infusion	3 - Misadventure
Y63.1	Incorrect dilution of fluid used during infusion	3 - Misadventure
Y63.2	Overdose of radiation given during therapy	3 - Misadventure
Y63.3	Inadvertent exposure of patient to radiation during medical care	3 - Misadventure
Y63.4	Failure in dosage in electroshock or insulin-shock therapy	3 - Misadventure
Y63.5	Inappropriate temperature in local application and packing	3 - Misadventure
Y63.6	Underdosing and nonadministration of necessary drug, medicament or biological substance	3 - Misadventure
Y63.8	Failure in dosage during other surgical and medical care	3 - Misadventure
Y63.9	Failure in dosage during unspecified surgical and medical care	3 - Misadventure
**Y64**	**Contaminated medical or biological substances**	3 - Misadventure
Y64.0	Contaminated medical or biological substance, transfused or infused	3 - Misadventure
Y64.1	Contaminated medical or biological substance, injected or used for immunization	3 - Misadventure
Y64.8	Contaminated medical or biological substance administered by other means	3 - Misadventure
Y64.9	Contaminated medical or biological substance administered by unspecified means	3 - Misadventure
**Y65**	**Other misadventures during surgical and medical care**	3 - Misadventure
Y65.0	Mismatched blood in transfusion	3 - Misadventure
Y65.1	Wrong fluid used in infusion	3 - Misadventure
Y65.2	Failure in suture or ligature during surgical operation	3 - Misadventure
Y65.3	Endotracheal tube wrongly placed during anesthetic procedure	3 - Misadventure
Y65.4	Failure to introduce or to remove other tube or instrument	3 - Misadventure
Y65.5	Performance of wrong procedure (operation)	3 - Misadventure
Y65.51	Performance of wrong procedure (operation) on correct patient	3 - Misadventure
Y65.52	Performance of procedure (operation) on patient not scheduled for surgery	3 - Misadventure
Y65.53	Performance of correct procedure (operation) on wrong side or body part	3 - Misadventure
Y65.8	Other specified misadventures during surgical and medical care	3 - Misadventure
**Y66**	**Nonadministration of surgical and medical care**	3 - Misadventure
**Y69**	**Unspecified misadventure during surgical and medical care**	3 - Misadventure
Y69.2		3 - Misadventure
Y69.9		3 - Misadventure
**Y70**	**Anesthesiology devices associated with adverse incidents**	5 - Medical or surgical devices
Y70.0	Diagnostic and monitoring anesthesiology devices associated with adverse incidents	5 - Medical or surgical devices
Y70.1	Therapeutic (nonsurgical) and rehabilitative anesthesiology devices associated with adverse incidents	5 - Medical or surgical devices
Y70.2	Prosthetic and other implants, materials and accessory anesthesiology devices associated with adverse incidents	5 - Medical or surgical devices
Y70.3	Surgical instruments, materials and anesthesiology devices (including sutures) associated with adverse incidents	5 - Medical or surgical devices
Y70.8	Miscellaneous anesthesiology devices associated with adverse incidents, not elsewhere classified	5 - Medical or surgical devices
**Y71**	**Cardiovascular devices associated with adverse incidents**	5 - Medical or surgical devices
Y71.0	Diagnostic and monitoring cardiovascular devices associated with adverse incidents	5 - Medical or surgical devices
Y71.1	Therapeutic (nonsurgical) and rehabilitative cardiovascular devices associated with adverse incidents	5 - Medical or surgical devices
Y71.2	Prosthetic and other implants, materials and accessory cardiovascular devices associated with adverse incidents	5 - Medical or surgical devices
Y71.3	Surgical instruments, materials and cardiovascular devices (including sutures) associated with adverse incidents	5 - Medical or surgical devices
Y71.8	Miscellaneous cardiovascular devices associated with adverse incidents, not elsewhere classified	5 - Medical or surgical devices
**Y72**	**Otorhinolaryngological devices associated with adverse incidents**	5 - Medical or surgical devices

© 2019 Sunshine JE et al. *JAMA Network Open*.

Y72.0	Diagnostic and monitoring otorhinolaryngological devices associated with adverse incidents	5 - Medical or surgical devices
Y72.1	Therapeutic (nonsurgical) and rehabilitative otorhinolaryngological devices associated with adverse incidents	5 - Medical or surgical devices
Y72.2	Prosthetic and other implants, materials and accessory otorhinolaryngological devices associated with adverse incidents	5 - Medical or surgical devices
Y72.3	Surgical instruments, materials and otorhinolaryngological devices (including sutures) associated with adverse incidents	5 - Medical or surgical devices
Y72.8	Miscellaneous otorhinolaryngological devices associated with adverse incidents, not elsewhere classified	5 - Medical or surgical devices
**Y73**	**Gastroenterology and urology devices associated with adverse incidents**	5 - Medical or surgical devices
Y73.0	Diagnostic and monitoring gastroenterology and urology devices associated with adverse incidents	5 - Medical or surgical devices
Y73.1	Therapeutic (nonsurgical) and rehabilitative gastroenterology and urology devices associated with adverse incidents	5 - Medical or surgical devices
Y73.2	Prosthetic and other implants, materials and accessory gastroenterology and urology devices associated with adverse incidents	5 - Medical or surgical devices
Y73.3	Surgical instruments, materials and gastroenterology and urology devices (including sutures) associated with adverse incidents	5 - Medical or surgical devices
Y73.8	Miscellaneous gastroenterology and urology devices associated with adverse incidents, not elsewhere classified	5 - Medical or surgical devices
**Y74**	**General hospital and personal-use devices associated with adverse incidents**	5 - Medical or surgical devices
Y74.0	Diagnostic and monitoring general hospital and personal-use devices associated with adverse incidents	5 - Medical or surgical devices
Y74.1	Therapeutic (nonsurgical) and rehabilitative general hospital and personal-use devices associated with adverse incidents	5 - Medical or surgical devices
Y74.2	Prosthetic and other implants, materials and accessory general hospital and personal-use devices associated with adverse incidents	5 - Medical or surgical devices
Y74.3	Surgical instruments, materials and general hospital and personal-use devices (including sutures) associated with adverse incidents	5 - Medical or surgical devices
Y74.8	Miscellaneous general hospital and personal-use devices associated with adverse incidents, not elsewhere classified	5 - Medical or surgical devices
**Y75**	**Neurological devices associated with adverse incidents**	5 - Medical or surgical devices
Y75.0	Diagnostic and monitoring neurological devices associated with adverse incidents	5 - Medical or surgical devices
Y75.1	Therapeutic (nonsurgical) and rehabilitative neurological devices associated with adverse incidents	5 - Medical or surgical devices
Y75.2	Prosthetic and other implants, materials and neurological devices associated with adverse incidents	5 - Medical or surgical devices
Y75.3	Surgical instruments, materials and neurological devices (including sutures) associated with adverse incidents	5 - Medical or surgical devices
Y75.8	Miscellaneous neurological devices associated with adverse incidents, not elsewhere classified	5 - Medical or surgical devices
**Y76**	**Obstetric and gynecological devices associated with adverse incidents**	5 - Medical or surgical devices
Y76.0	Diagnostic and monitoring obstetric and gynecological devices associated with adverse incidents	5 - Medical or surgical devices
Y76.1	Therapeutic (nonsurgical) and rehabilitative obstetric and gynecological devices associated with adverse incidents	5 - Medical or surgical devices
Y76.2	Prosthetic and other implants, materials and accessory obstetric and gynecological devices associated with adverse incidents	5 - Medical or surgical devices
Y76.3	Surgical instruments, materials and obstetric and gynecological devices (including sutures) associated with adverse incidents	5 - Medical or surgical devices
Y76.8	Miscellaneous obstetric and gynecological devices associated with adverse incidents, not elsewhere classified	5 - Medical or surgical devices
Y76.9	Obstetric and gynaecological devices associated with adverse incidents	5 - Medical or surgical devices
**Y77**	**Ophthalmic devices associated with adverse incidents**	5 - Medical or surgical devices
Y77.0	Diagnostic and monitoring ophthalmic devices associated with adverse incidents	5 - Medical or surgical devices

© 2019 Sunshine JE et al. *JAMA Network Open*.

Y77.1	Therapeutic (nonsurgical) and rehabilitative ophthalmic devices associated with adverse incidents	5 - Medical or surgical devices
Y77.2	Prosthetic and other implants, materials and accessory ophthalmic devices associated with adverse incidents	5 - Medical or surgical devices
Y77.3	Surgical instruments, materials and ophthalmic devices (including sutures) associated with adverse incidents	5 - Medical or surgical devices
Y77.8	Miscellaneous ophthalmic devices associated with adverse incidents, not elsewhere classified	5 - Medical or surgical devices
**Y78**	**Radiological devices associated with adverse incidents**	5 - Medical or surgical devices
Y78.0	Diagnostic and monitoring radiological devices associated with adverse incidents	5 - Medical or surgical devices
Y78.1	Therapeutic (nonsurgical) and rehabilitative radiological devices associated with adverse incidents	5 - Medical or surgical devices
Y78.2	Prosthetic and other implants, materials and accessory radiological devices associated with adverse incidents	5 - Medical or surgical devices
Y78.3	Surgical instruments, materials and radiological devices (including sutures) associated with adverse incidents	5 - Medical or surgical devices
Y78.8	Miscellaneous radiological devices associated with adverse incidents, not elsewhere classified	5 - Medical or surgical devices
**Y79**	**Orthopedic devices associated with adverse incidents**	5 - Medical or surgical devices
Y79.0	Diagnostic and monitoring orthopedic devices associated with adverse incidents	5 - Medical or surgical devices
Y79.1	Therapeutic (nonsurgical) and rehabilitative orthopedic devices associated with adverse incidents	5 - Medical or surgical devices
Y79.2	Prosthetic and other implants, materials and accessory orthopedic devices associated with adverse incidents	5 - Medical or surgical devices
Y79.3	Surgical instruments, materials and orthopedic devices (including sutures) associated with adverse incidents	5 - Medical or surgical devices
Y79.8	Miscellaneous orthopedic devices associated with adverse incidents, not elsewhere classified	5 - Medical or surgical devices
**Y80**	**Physical medicine devices associated with adverse incidents**	5 - Medical or surgical devices
Y80.0	Diagnostic and monitoring physical medicine devices associated with adverse incidents	5 - Medical or surgical devices
Y80.1	Therapeutic (nonsurgical) and rehabilitative physical medicine devices associated with adverse incidents	5 - Medical or surgical devices
Y80.2	Prosthetic and other implants, materials and accessory physical medicine devices associated with adverse incidents	5 - Medical or surgical devices
Y80.3	Surgical instruments, materials and physical medicine devices (including sutures) associated with adverse incidents	5 - Medical or surgical devices
Y80.8	Miscellaneous physical medicine devices associated with adverse incidents, not elsewhere classified	5 - Medical or surgical devices
**Y81**	**General- and plastic-surgery devices associated with adverse incidents**	5 - Medical or surgical devices
Y81.0	Diagnostic and monitoring general- and plastic-surgery devices associated with adverse incidents	5 - Medical or surgical devices
Y81.1	Therapeutic (nonsurgical) and rehabilitative general- and plastic-surgery devices associated with adverse incidents	5 - Medical or surgical devices
Y81.2	Prosthetic and other implants, materials and accessory general- and plastic-surgery devices associated with adverse incidents	5 - Medical or surgical devices
Y81.3	Surgical instruments, materials and general- and plastic-surgery devices (including sutures) associated with adverse incidents	5 - Medical or surgical devices
Y81.8	Miscellaneous general- and plastic-surgery devices associated with adverse incidents, not elsewhere classified	5 - Medical or surgical devices
**Y82**	**Other and unspecified medical devices associated with adverse incidents**	5 - Medical or surgical devices
Y82.0	Diagnostic and monitoring devices	5 - Medical or surgical devices
Y82.1	Therapeutic (nonsurgical) and rehabilitative devices	5 - Medical or surgical devices
Y82.2	Prosthetic and other implants, materials and accessory devices	5 - Medical or surgical devices

© 2019 Sunshine JE et al. *JAMA Network Open*.

Y82.3	Surgical instruments, materials and devices (including sutures)	5 - Medical or surgical devices
Y82.8	Other medical devices associated with adverse incidents	5 - Medical or surgical devices
Y82.9	Unspecified medical devices associated with adverse incidents	5 - Medical or surgical devices
**Y83**	**Surgical operation and other surgical procedures as the cause of abnormal reaction of the patient, or of later complication, without mention of misadventure at the time of the procedure**	2 - Surgical and peri-operative adverse events
Y83.0	Surgical operation with transplant of whole organ as the cause of abnormal reaction of the patient, or of later complication, without mention of misadventure at the time of the procedure	2 - Surgical and peri-operative adverse events
Y83.1	Surgical operation with implant of artificial internal device as the cause of abnormal reaction of the patient, or of later complication, without mention of misadventure at the time of the procedure	5 - Medical or surgical devices
Y83.2	Surgical operation with anastomosis, bypass or graft as the cause of abnormal reaction of the patient, or of later complication, without mention of misadventure at the time of the procedure	2 - Surgical and peri-operative adverse events
Y83.3	Surgical operation with formation of external stoma as the cause of abnormal reaction of the patient, or of later complication, without mention of misadventure at the time of the procedure	2 - Surgical and peri-operative adverse events
Y83.4	Other reconstructive surgery as the cause of abnormal reaction of the patient, or of later complication, without mention of misadventure at the time of the procedure	2 - Surgical and peri-operative adverse events
Y83.5	Amputation of limb(s) as the cause of abnormal reaction of the patient, or of later complication, without mention of misadventure at the time of the procedure	2 - Surgical and peri-operative adverse events
Y83.6	Removal of other organ (partial) (total) as the cause of abnormal reaction of the patient, or of later complication, without mention of misadventure at the time of the procedure	2 - Surgical and peri-operative adverse events
Y83.8	Other surgical procedures as the cause of abnormal reaction of the patient, or of later complication, without mention of misadventure at the time of the procedure	2 - Surgical and peri-operative adverse events
Y83.9	Surgical procedure, unspecified as the cause of abnormal reaction of the patient, or of later complication, without mention of misadventure at the time of the procedure	2 - Surgical and peri-operative adverse events
**Y84**	**Other medical procedures as the cause of abnormal reaction of the patient, or of later complication, without mention of misadventure at the time of the procedure**	4 - Medical management
Y84.0	Cardiac catheterization as the cause of abnormal reaction of the patient, or of later complication, without mention of misadventure at the time of the procedure	2 - Surgical and peri-operative adverse events
Y84.1	Kidney dialysis as the cause of abnormal reaction of the patient, or of later complication, without mention of misadventure at the time of the procedure	4 - Medical management
Y84.2	Radiological procedure and radiotherapy as the cause of abnormal reaction of the patient, or of later complication, without mention of misadventure at the time of the procedure	4 - Medical management
Y84.3	Shock therapy as the cause of abnormal reaction of the patient, or of later complication, without mention of misadventure at the time of the procedure	4 - Medical management
Y84.4	Aspiration of fluid as the cause of abnormal reaction of the patient, or of later complication, without mention of misadventure at the time of the procedure	4 - Medical management
Y84.5	Insertion of gastric or duodenal sound as the cause of abnormal reaction of the patient, or of later complication, without mention of misadventure at the time of the procedure	4 - Medical management
Y84.6	Urinary catheterization as the cause of abnormal reaction of the patient, or of later complication, without mention of misadventure at the time of the procedure	4 - Medical management
Y84.7	Blood-sampling as the cause of abnormal reaction of the patient, or of later complication, without mention of misadventure at the time of the procedure	4 - Medical management
Y84.8	Other medical procedures as the cause of abnormal reaction of the patient, or of later complication, without mention of misadventure at the time of the procedure	4 - Medical management
Y84.9	Medical procedure, unspecified as the cause of abnormal reaction of the patient, or of later complication, without mention of misadventure at the time of the procedure	4 - Medical management
**Y88**	**Sequelae with surgical and medical care as external cause**	2 - Surgical and peri-operative adverse events
Y88.0	Sequelae of adverse effects caused by drugs, medicaments and biological substances in therapeutic use	1 - Adverse drug events
Y88.1	Sequelae of misadventures to patients during surgical and medical procedures	3 - Misadventure
Y88.2	Sequelae of adverse incidents associated with medical devices in diagnostic and therapeutic use	5 - Medical or surgical devices
Y88.3	Sequelae of surgical and medical procedures as the cause of abnormal reaction of the patient, or of later complication, without mention of misadventure at the time of the procedure	2 - Surgical and peri-operative adverse events

© 2019 Sunshine JE et al. *JAMA Network Open*.

**eTable 3.** Selected cause-of-death ensemble modeling (CODEm) covariates used to generate the adverse effects of medical harm (AEMT) models.	
**Covariate**	**level**
Health system access quality index	2
Lag distribution income per capita	3
Sociodemographic index	3

**eTable 4.** Result of redistrbution of Garbage codes on AEMT as underlying cause by different non underlying cause by ICD9 and ICD10		
**Underlying Cause**	**ICD10**	**ICD9**
Original (raw data)	74.1%	81.1%
Sepsis	5.8%	3.1%
Hyperkalemia	2.7%	2.2%
Atrial flutter/fibrillation	2.7%	1.5%
Peritonitis	2.5%	1.3%
Osteomyelitis	1.9%	1.2%
Arrhythmia	1.4%	1.1%
Unspecified factor X59	1.4%	1.1%
Renal failure	1.3%	1.0%
Shock and cardiac arrest	0.9%	0.9%
All, ill-defined	0.9%	0.9%
External causes	0.9%	0.6%
Anemia unspecified	0.5%	0.5%
Arterial embolism	0.5%	0.5%
Toxic encephalopathy	0.5%	0.5%
Pyothorax	0.3%	0.4%
Pneumonitis	0.3%	0.3%
Pleurisy	0.3%	0.2%
All garbage package	0.2%	0.2%
Cerebral palsy	0.2%	0.2%
Asphyxia and hypoxemia	0.2%	0.2%
USA Armed Forces accident	0.1%	0.2%
Urinary obstruction	0.1%	0.1%
Fistula	0.1%	0.1%
Cerebral edema	0.1%	0.1%
Other	0.2%	0.1%

© 2019 Sunshine JE et al. *JAMA Network Open*.

eTable 5. Frequency of causes of death in the cause-of-death (COD) chain when Adverse Effects of Medical Treatment (AEMT) is the underlying cause of death, US 1980-2014.

		AEMT by type							
	Non-underlying causes of death	Adverse drug events	Surgical and peri-operative adverse events	Misadventure	Medical management	Medical or surgical devices	Other	All types of AEMT per 1000	Death totals of other causes in the chain when AEMT is the underlying cause of death
External cause of Injuries	All External cause of Injuries	464.74	307.25	325.17	391.34	960.72	127.21	363.02	39,620
	Specific anatomic injuries (ICD N-codes)	402.27	280.76	258.11	373.78	947.28	111.31	331.76	36,209
	Unintentional injuries other	14.43	11.31	24.39	10.81	3.66	8.83	12.28	1,340
	Transport injuries	3.30	5.97	18.16	2.62	0.61	0.00	6.03	658
	Falls	5.15	5.20	4.62	0.92	9.16	5.30	4.73	516
	Interpersonal violence	33.40	1.83	3.65	0.26	0.00	0.00	4.48	489
	Self-harm	5.57	1.95	13.86	2.62	0.00	1.77	3.29	359
	Fire, heat, and hot substances	0.62	0.23	2.36	0.33	0.00	0.00	0.45	49
Chronic infectious disease	All Chronic infectious disease	7.53	3.65	7.31	5.18	1.63	1.77	4.42	482
	Tuberculosis	1.13	1.31	2.90	1.24	0.20	0.00	1.37	149
	Fever Unknown	4.43	0.86	1.07	1.97	0.61	0.00	1.34	146
	CNS Abscess	1.24	0.82	1.29	1.57	0.61	1.77	1.00	109
	Salpingitis	0.00	0.49	1.72	0.13	0.20	0.00	0.49	53
	Syphilis	0.62	0.07	0.11	0.20	0.00	0.00	0.14	15
	Gonococcal infection	0.00	0.01	0.21	0.00	0.00	0.00	0.03	3
	Cerebral Cysts	0.00	0.03	0.00	0.00	0.00	0.00	0.02	2
	Other sexually transmitted diseases	0.00	0.03	0.00	0.00	0.00	0.00	0.02	2
	Cysticercosis	0.00	0.01	0.00	0.00	0.00	0.00	0.01	1
	Unspecified Parasitic D	0.10	0.00	0.00	0.00	0.00	0.00	0.01	1
	Genital herpes	0.00	0.00	0.00	0.07	0.00	0.00	0.01	1
Congenital birth defects	All Congenital birth defects	8.04	9.45	27.51	10.16	4.88	7.07	10.75	1,173
	Congenital heart anomalies	2.27	3.13	14.83	2.56	1.83	3.53	3.91	427
	Other congenital birth defects	2.47	1.59	4.84	3.21	0.81	0.00	2.13	232
	Congenital musculoskeletal and limb anomalies	0.82	1.50	1.72	1.24	1.22	1.77	1.41	154
	Down syndrome	0.52	1.12	1.18	1.05	0.20	0.00	1.02	111
	Digestive congenital anomalies	0.41	0.79	2.58	0.79	0.20	0.00	0.88	96
	Neural tube defects	0.41	0.56	1.07	0.52	0.00	0.00	0.56	61
	Other chromosomal abnormalities	0.31	0.42	0.21	0.46	0.20	0.00	0.38	42
	Unspecified Congenital diseases	0.21	0.22	0.11	0.20	0.00	1.77	0.20	22
	Urogenital congenital anomalies	0.62	0.04	0.97	0.07	0.00	0.00	0.17	19
	Turner syndrome	0.00	0.03	0.00	0.00	0.41	0.00	0.04	4

© 2019 Sunshine JE et al. *JAMA Network Open*.

	Cleft lip and cleft palate	0.00	0.04	0.00	0.07	0.00	0.00	0.04	4
	Klinefelter syndrome	0.00	0.01	0.00	0.00	0.00	0.00	0.01	1
Digestive diseases	All Digestive diseases	111.75	237.50	178.49	130.71	79.99	219.08	199.17	21,738
	Paralytic ileus and intestinal obstruction	2.68	65.34	11.07	7.34	6.31	1.77	44.04	4,807
	Unspecified Digestive Diseases	9.48	31.53	20.85	29.75	8.55	22.97	27.33	2,983
	Vascular intestinal disorders	3.92	38.09	8.70	5.50	6.51	42.40	26.59	2,902
	Cirrhosis and other chronic liver diseases	38.35	21.80	43.95	27.06	22.19	33.57	25.98	2,835
	Other digestive diseases	2.99	23.92	8.60	6.75	1.22	3.53	17.23	1,880
	Gastrointestinal Bleeding	24.85	13.94	15.37	19.59	12.42	68.90	16.03	1,750
	Peptic ulcer disease	9.38	8.68	9.35	7.60	3.46	3.53	8.38	915
	Gallbladder and biliary diseases	3.20	6.77	17.52	3.34	4.07	10.60	6.79	741
	Inguinal, femoral, and abdominal hernia	0.72	6.60	3.12	0.79	1.42	0.00	4.70	513
	Inflammatory bowel disease	5.46	3.96	4.84	6.49	3.46	14.13	4.55	497
	Gastroesophageal reflux disease	2.68	3.26	12.14	4.65	3.46	3.53	4.17	455
	Pancreatitis	4.23	3.30	5.91	3.08	1.63	5.30	3.51	383
	Ascites	0.72	3.66	3.98	3.93	1.02	0.00	3.33	363
	Diverticular disease of intestines	0.62	3.47	7.09	1.70	2.24	5.30	3.23	353
	Appendicitis	0.31	1.41	2.26	0.66	0.20	0.00	1.22	133
	Hemorrhoids, anal fissure, anal abscess, and fistula	0.52	0.82	2.36	1.44	0.61	1.77	1.01	110
	Gastritis and duodenitis	1.44	0.71	0.64	0.79	1.02	1.77	0.80	87
	Liver Abscess	0.10	0.19	0.64	0.26	0.20	0.00	0.23	25
	Celiac disease	0.10	0.06	0.11	0.00	0.00	0.00	0.05	6
Musculoskeletal disorders	Musculoskeletal disorders	23.51	10.78	10.21	14.35	16.08	21.20	12.65	1,381
acute infectious diseases	All acute infectious diseases	24.74	15.35	14.29	34.27	21.37	28.27	19.08	2,082
	Unspecified Infectious diseases	6.80	6.50	1.93	12.64	12.01	8.83	7.26	792
	opportunistic infectious	11.75	4.25	2.90	13.56	4.27	8.83	6.13	669
	Other Infectious diseases	3.09	2.08	2.04	5.44	2.85	5.30	2.68	293
	Hepatitis	1.96	1.12	5.37	1.31	1.02	5.30	1.60	175
	Meningitis	1.13	1.40	2.04	1.31	1.22	0.00	1.40	153
Urogenital diseases	All Urogenital diseases	34.64	52.04	71.89	65.19	24.63	63.60	52.85	5,768
	Urinary diseases	33.81	38.45	67.81	63.81	24.22	63.60	43.58	4,756
	Gynecological diseases	0.82	13.59	4.08	1.38	0.41	0.00	9.27	1,012
Endocrine and metabolic disorders	All Endocrine and metabolic disorders	88.14	50.78	39.97	38.59	70.63	95.41	52.60	5,741
	Obesity	17.53	25.13	12.47	10.42	29.51	12.37	21.45	2,341
	Other endocrine, metabolic, blood, and immune disorders	56.60	14.76	20.74	17.49	15.67	61.84	19.65	2,145
	Lipoprotein metabolism and	6.19	4.71	2.15	3.08	14.45	12.37	4.87	532

	other lipidaemias disorders								
	Hypothyroidism	5.67	2.98	1.72	3.01	10.38	5.30	3.46	378
	Unspecified Endocrine&Metabolic Diseases	1.24	2.03	1.18	2.95	0.20	1.77	1.93	211
	Unspecified Immunodeficiency	0.52	0.43	1.40	1.18	0.00	0.00	0.60	66
	Cystic fibrosis	0.10	0.42	0.21	0.13	0.00	1.77	0.32	35
	Hyperthyroidism	0.31	0.32	0.11	0.33	0.41	0.00	0.30	33
Blood Disorders	All Blood Disorders	22.27	14.57	13.22	26.34	28.09	107.77	17.88	1,951
	Anemia	15.26	13.32	11.39	21.62	26.66	65.37	15.36	1,676
	G6PD deficiency	5.88	0.88	1.40	3.73	1.42	15.90	1.87	204
	Thalassemias	0.82	0.27	0.43	0.52	0.00	3.53	0.38	41
	Unspecified Blood D	0.31	0.09	0.00	0.39	0.00	22.97	0.26	28
	Sickle cell disorders	0.00	0.01	0.00	0.07	0.00	0.00	0.02	2
Sense organ diseases	Sense organ diseases	0.00	0.03	0.00	0.00	0.00	0.00	0.02	2
Diarrheal diseases	Diarrheal diseases	4.64	1.97	1.18	2.69	5.09	15.90	2.46	268
Impossible cause for death	impossible cause for death	164.43	240.70	65.44	173.03	708.12	173.14	230.21	25,125
Skin Diseases	Skin Diseases	35.67	23.55	6.23	30.20	17.91	8.83	23.75	2,592
Maternal disorders	Maternal disorders	1.86	0.39	3.87	0.46	0.41	0.00	0.82	90
Mental disorders	All Mental disorders	49.59	11.17	29.23	14.15	8.75	14.13	16.45	1,795
	Drug use disorders	31.34	4.22	10.85	5.44	1.42	3.53	7.24	790
	Alcohol use disorders	14.74	5.16	16.66	6.16	5.70	8.83	7.17	783
	Mental disorders	3.51	1.79	1.72	2.56	1.63	1.77	2.03	222
Kidney disease	kidney disease	126.29	92.96	80.92	110.07	126.20	219.08	99.44	10,853
Nutritional deficiencies	Nutritional deficiencies	6.91	16.79	8.60	23.59	15.67	12.37	16.09	1,756
Chronic intermediate and immediate cause	All Chronic intermediate and immediate cause	31.65	47.63	32.13	71.41	39.69	28.27	47.75	5,212
	Pneumonitis	15.88	18.72	10.96	31.25	14.86	7.07	19.32	2,109
	Senility	4.02	10.07	2.36	11.40	10.58	5.30	9.06	989
	different type of Paralysis	3.20	6.34	3.44	11.33	2.65	0.00	6.31	689
	Respiratory Failure Chronic	2.89	5.00	7.95	5.31	1.22	7.07	4.95	540
	Cachexia	2.27	3.18	2.47	6.16	3.26	1.77	3.45	377
	Osteomyelitis	1.86	1.71	1.50	1.57	3.87	0.00	1.78	194
	Cerebral Palsy	0.93	1.41	2.36	2.62	1.22	1.77	1.61	176
	Hydrocephalus	0.31	0.58	0.00	0.66	1.42	0.00	0.55	60
	Fistula	0.10	0.39	0.32	0.66	0.00	0.00	0.38	41
	Amyloidosis	0.21	0.22	0.75	0.46	0.61	5.30	0.34	37
Cardiovascular diseases	All Cardiovascular diseases	462.27	467.05	553.41	438.31	660.70	818.02	480.51	52,443
	Ischemic heart disease	115.05	107.53	139.26	87.07	145.94	157.24	110.03	12,009
	Heart failure	59.48	72.23	54.05	67.16	82.43	70.67	69.29	7,562
	Hypertension	55.88	51.85	32.45	50.12	108.69	90.11	53.07	5,792
	Cerebrovascular disease	59.69	40.88	28.15	70.17	38.47	183.75	46.20	5,042
	Pulmonary Embolism	14.43	44.86	12.14	13.23	53.33	75.97	35.49	3,873
	Unspecified CVD	25.46	30.32	58.46	23.52	37.66	33.57	31.68	3,458
	Atrial fibrillation and flutter	27.32	21.67	9.13	19.98	50.27	24.73	22.17	2,420
	Other cardiovascular and circulatory diseases	9.59	12.09	62.76	13.63	9.16	14.13	16.28	1,777
	Atherosclerosis	8.76	12.51	20.20	12.45	4.48	3.53	12.42	1,355

© 2019 Sunshine JE et al. *JAMA Network Open*.

	Nonrheumatic valvular disorders	10.82	10.16	22.78	8.06	39.49	22.97	12.39	1,352
	Peripheral artery disease	8.97	12.83	7.31	10.81	8.55	19.43	11.57	1,263
	Hypertensive heart disease	16.80	8.20	31.06	11.40	8.55	1.77	11.34	1,238
	Cardiovascular diseases	10.52	9.25	15.04	14.87	8.14	10.60	10.60	1,157
	Cardiomyopathy and myocarditis	16.19	6.86	11.82	11.47	12.82	8.83	9.03	986
	Aortic aneurysm	3.40	6.85	16.44	3.93	3.87	5.30	6.81	743
	Phlebitis and thrombophlebitis	8.04	6.04	5.27	5.77	13.23	67.14	6.75	737
	Rheumatic heart disease	4.33	4.74	16.98	3.34	14.25	12.37	6.02	657
	Endocarditis	4.74	4.52	6.02	8.06	18.93	5.30	5.82	635
	Arterial Embolism	2.47	3.49	3.87	2.95	2.04	10.60	3.33	363
	Varicose veins	0.31	0.17	0.21	0.33	0.41	0.00	0.22	24
Neoplasm	All neoplasm	74.33	54.96	153.13	86.03	43.56	100.71	69.12	7,544
	Cancer with Unspecified site	13.51	10.48	32.77	16.44	6.31	14.13	13.31	1,453
	Colon and rectum cancer	5.15	8.60	18.70	5.77	4.48	1.77	8.54	932
	Tracheal, bronchus, and lung cancer	9.48	5.61	27.40	7.80	3.26	1.77	7.99	872
	Leukemia	7.63	2.62	7.52	11.20	2.85	38.87	4.88	533
	Breast cancer	6.70	3.69	5.70	6.09	5.29	1.77	4.53	494
	Other neoplasms	3.81	3.39	6.23	4.85	3.26	26.50	3.99	435
	Prostate cancer	3.20	3.23	5.16	6.55	5.90	0.00	3.96	432
	Non-Hodgkin lymphoma	7.84	1.82	3.98	6.09	2.04	7.07	3.17	346
	Brain and nervous system cancer	2.47	2.06	3.76	2.03	0.81	0.00	2.17	237
	Bladder cancer	1.86	1.86	3.12	1.77	1.42	1.77	1.93	211
	Kidney cancer	1.96	1.24	3.98	1.70	1.02	1.77	1.59	174
	Pancreatic cancer	0.93	0.99	4.84	1.70	0.41	1.77	1.39	152
	Esophageal cancer	0.21	1.15	5.05	1.24	0.81	0.00	1.39	152
	Multiple myeloma	2.06	0.71	2.58	2.36	1.22	3.53	1.26	137
	Ovarian cancer	1.03	1.10	2.47	1.11	0.81	0.00	1.19	130
	Stomach cancer	0.52	1.04	3.12	1.11	0.41	0.00	1.15	125
	Larynx cancer	0.41	0.98	2.15	0.79	0.61	0.00	0.98	107
	Liver cancer	0.82	0.52	3.65	1.18	0.20	0.00	0.89	97
	Lip and oral cavity cancer	0.62	0.69	1.50	0.92	0.41	0.00	0.77	84
	Cervical cancer	0.21	0.53	2.58	1.18	0.41	0.00	0.76	83
	Uterine cancer	0.21	0.71	1.07	0.59	0.41	0.00	0.66	72
	Non-melanoma skin cancer	0.62	0.49	0.54	0.66	0.20	0.00	0.51	56
	Hodgkin lymphoma	1.24	0.27	1.07	0.92	0.20	0.00	0.51	56
	Malignant skin melanoma	0.82	0.27	0.64	0.52	0.41	0.00	0.39	43
	Other pharynx cancer	0.10	0.26	1.40	0.39	0.00	0.00	0.35	38
	Thyroid cancer	0.31	0.24	0.97	0.33	0.41	0.00	0.33	36
	Gallbladder and biliary tract cancer	0.10	0.33	0.64	0.26	0.00	0.00	0.31	34
	Testicular cancer	0.31	0.04	0.21	0.20	0.00	0.00	0.10	11
	Nasopharynx cancer	0.10	0.04	0.32	0.20	0.00	0.00	0.09	10
	Mesothelioma	0.10	0.00	0.00	0.07	0.00	0.00	0.02	2
Diabetes and diabetes related disorders	Diabetes and diabetes Related disorders	69.59	66.53	43.63	71.28	89.76	91.87	66.69	7,279

© 2019 Sunshine JE et al. *JAMA Network Open*.

Acute intermediate and immediate cause	All Acute intermediate and immediate cause	609.90	718.44	590.80	748.21	638.92	602.47	697.90	76,169
	Sepsis	75.15	210.17	111.97	249.10	248.52	164.31	196.73	21,471
	Shock& Cardiac Arrest	181.55	198.09	95.85	160.91	166.09	185.51	181.20	19,776
	Asphyxia & Hypoxemia	79.07	88.45	50.61	86.61	38.27	33.57	81.59	8,905
	Respiratory Failure Acute	78.35	67.33	28.37	77.44	90.17	123.67	67.72	7,391
	Coma & Stupor	25.57	44.36	60.93	50.06	1.22	1.77	42.73	4,664
	Peritonitis	3.81	28.94	94.56	26.01	3.46	5.30	30.62	3,342
	Dehydration and Acid-Base disorders	29.59	23.65	15.04	28.17	19.54	30.04	23.92	2,611
	Flutter, Fibrillation, Arrhythmia	20.31	14.08	12.68	13.17	32.57	17.67	15.24	1,663
	Brain Anoxia	44.74	8.37	8.38	14.74	17.10	10.60	12.90	1,408
	Hemorrhage, Unspecified	2.78	5.92	70.92	8.12	0.00	0.00	11.20	1,222
	Convulsions	25.88	7.75	4.30	14.48	4.68	1.77	9.84	1,074
	Pulmonary Edema	16.49	7.23	6.13	7.60	6.92	5.30	7.99	872
	Acute Abdomen	4.43	8.52	2.69	1.64	0.81	1.77	6.31	689
	Hemothorax	0.62	0.52	23.21	1.64	1.42	0.00	2.66	290
	Encephalopathy	2.68	1.10	0.32	2.69	2.44	3.53	1.47	160
	Hepatic Failure	6.29	0.69	1.61	0.92	0.41	7.07	1.32	144
	Nausea And Vomiting	2.27	0.98	0.43	1.18	0.41	0.00	1.04	114
	Compression of Brain	2.68	0.65	0.64	0.85	1.22	3.53	0.90	98
	Cerebral Edema	1.86	0.69	0.54	0.52	1.83	3.53	0.82	90
	Pulmonary Collapse	2.16	0.48	0.43	0.66	0.41	0.00	0.64	70
	Intracranial Hypertension	0.41	0.35	0.21	0.26	0.61	1.77	0.35	38
	Sudden infant death syndrome	0.93	0.01	0.00	1.18	0.00	0.00	0.26	28
	Toxic liver disease	1.44	0.01	0.00	0.07	0.00	0.00	0.15	16
	Interstitial Emphysema	0.10	0.04	0.86	0.07	0.00	0.00	0.12	13
	Toxic Encephalopathy	0.41	0.07	0.00	0.00	0.20	0.00	0.09	10
	Acquired hemolytic anemia	0.31	0.00	0.11	0.13	0.61	1.77	0.09	10
Neurological disorders	All Neurological disorders	63.40	59.16	54.37	98.47	76.74	40.64	65.32	7,129
	Alzheimer disease and other dementias	17.22	31.66	14.83	58.25	57.40	28.27	33.80	3,689
	Unspecified neurological disorders	33.20	16.72	27.72	19.07	5.70	5.30	18.89	2,062
	Parkinson disease	4.12	5.26	3.33	11.47	10.18	1.77	6.07	662
	Other neurological disorders	4.74	3.85	6.23	5.63	2.24	5.30	4.32	471
	Epilepsy	2.89	0.86	1.61	1.38	0.81	0.00	1.17	128
	Multiple sclerosis	1.24	0.81	0.64	2.69	0.41	0.00	1.07	117
Chronic respiratory diseases	All Chronic respiratory diseases	125.67	79.36	97.79	85.37	77.55	72.44	85.77	9,361
	Chronic obstructive pulmonary disease	49.79	45.97	47.93	43.70	48.85	45.94	46.29	5,052
	Unspecified chronic respiratory diseases	21.44	15.49	13.65	17.62	9.16	10.60	15.85	1,730
	Other chronic respiratory diseases	13.81	2.52	12.57	6.68	0.20	0.00	4.85	529
	Asthma	20.21	3.03	2.69	3.41	5.09	1.77	4.66	509
	Interstitial lung disease and pulmonary sarcoidosis	10.10	3.01	5.59	4.98	5.70	8.83	4.29	468

© 2019 Sunshine JE et al. *JAMA Network Open*.

	Chronic diseases of upper respiratory system	4.33	4.64	2.36	0.59	0.00	0.00	3.62	395
	Pneumothorax	1.55	1.74	11.39	4.72	1.22	1.77	2.94	321
	Chronic respiratory diseases	3.61	1.82	0.43	2.03	6.31	3.53	2.10	229
	Pneumoconiosis	0.41	0.66	0.21	0.72	0.61	0.00	0.60	66
	Prothorax	0.41	0.48	0.97	0.92	0.41	0.00	0.57	62
HIV	HIV	4.23	0.81	4.41	4.00	0.41	1.77	1.85	202
Acute respiratory infections	acute respiratory infections	62.27	51.94	85.54	71.94	46.20	91.87	58.47	6,381
Neonatal disorders	Neonatal disorders	6.19	2.28	15.04	11.86	0.00	15.90	5.02	548
Poisoning and overdoses	Poisoning and overdoses	6.29	1.25	13.86	2.42	0.61	0.00	2.90	317
Adverse effects of medical treatment		999.59	984.59	999.89	999.93	999.59	1000.00	990.13	108,064
Total	**Grand Total**	4008.87	4152.02	4264.24	4267.77	4763.89	3978.80	4191.70	403,066

eTable 6. Number of each subtype of AEMT by year where AEMT was certified as the underlying cause of death, 1980 to 2014, both sexes combined

Year	AEMT by type						Total
	Adverse drug events	Surgical and peri-operative adverse events	Misadventure	Medical management	Medical or surgical devices	Other	
1980	165	1,970	213	314	2	0	2,664
1981	210	2,026	230	305	2	2	2,775
1982	162	2,182	241	241	0	0	2,826
1983	170	2,085	284	321	1	1	2,862
1984	166	2,034	274	341	1	2	2,818
1985	179	2,176	260	425	4	2	3,046
1986	198	2,312	321	413	0	2	3,246
1987	154	2,281	371	417	0	2	3,225
1988	181	2,234	329	468	2	1	3,215
1989	149	2,169	410	462	1	0	3,191
1990	169	2,030	418	414	6	2	3,039
1991	169	1,849	382	430	1	1	2,832
1992	162	2,034	435	421	1	0	3,053
1993	201	2,084	418	429	0	0	3,132

© 2019 Sunshine JE et al. *JAMA Network Open*.

1994	174	1,980	409	429	0	1	2,993
1995	209	2,030	450	439	1	0	3,129
1996	253	2,126	465	559	0	1	3,404
1997	248	2,272	526	492	0	0	3,538
1998	279	2,363	530	578	0	2	3,752
1999	274	1,894	179	492	299	12	3,150
2000	292	2,166	183	502	300	27	3,470
2001	308	2,087	177	465	336	14	3,387
2002	282	2,030	169	434	313	22	3,250
2003	362	1,992	166	425	352	26	3,323
2004	398	1,865	171	495	335	26	3,290
2005	371	1,760	147	451	309	58	3,096
2006	379	1,626	167	436	295	45	2,948
2007	378	1,721	155	461	293	56	3,064
2008	465	1,646	129	450	304	46	3,040
2009	434	1,741	142	417	325	35	3,094
2010	450	1,625	134	411	282	38	2,940
2011	435	1,704	112	484	277	36	3,048
2012	434	1,671	103	514	291	37	3,050
2013	418	1,903	112	470	305	38	3,246
2014	422	1,725	94	458	275	31	3,005
**Total: 1980-2014**	**9,700**	**69,393**	**9,306**	**15,263**	**4,913**	**566**	**109,141**
**Total %: 1980-2014**	**8.9%**	**63.6%**	**8.5%**	**14.0%**	**4.5%**	**0.5%**	

© 2019 Sunshine JE et al. *JAMA Network Open*.

eTable 7. Rate of occurrence of AEMT in the cause-of death chain by other cause category (per 1,000 deaths in each category) when AEMT was NOT certified as the underlying cause of death, 1980 to 2014, both sexes combined

Underlying causes of death	AEMT by type						Any type of AEMT per 1000 deaths in category	Death totals with AEMT in cause-of-death chain, but not underlying cause.
	Adverse drug events	Surgical and peri-operative adverse events	Misadventure	Medical management	Medical or surgical devices	Other		
External cause of Injuries	29.76	5.69	115.29	51.74	0.47	0.03	202.98	1,028,430
Chronic infectious disease	12.57	34.97	0.34	3.71	0.17	0.18	51.95	3,944
Congenital birth defects	6.55	39.17	0.50	2.55	0.84	0.07	49.68	18,940
Digestive diseases	9.85	35.79	0.69	2.51	0.17	0.39	49.40	141,744
Musculoskeletal disorders	20.80	12.36	0.26	2.74	1.63	0.74	38.53	10,016
acute infectious diseases	13.09	12.91	4.07	7.08	0.27	0.24	37.66	12,353
Urogenital diseases	5.96	20.62	0.33	5.55	0.29	0.10	32.86	19,413
Endocrine and metabolic disorders	11.37	16.90	0.54	2.80	0.32	0.74	32.68	16,848
Blood Disorders	9.03	5.76	0.45	10.42	0.30	1.90	27.86	4,053
Sense organ diseases	10.87	5.43	0.00	10.87	0.00	0.00	27.17	5
Diarrheal diseases	14.13	8.76	0.20	2.00	0.72	0.26	26.06	3,013
Impossible cause for death	11.51	11.04	0.16	1.49	1.29	0.02	25.51	25,621
Skin Diseases	8.70	12.64	0.20	2.42	0.33	0.13	24.42	3,122
Maternal disorders	6.08	11.93	1.59	2.95	0.68	0.00	23.23	409
Mental disorders	19.05	1.62	0.32	0.83	0.12	0.20	22.14	7,922
Kidney disease	3.01	9.02	0.28	5.79	0.27	0.25	18.61	29,664
Nutritional deficiencies	7.52	6.24	0.22	3.59	0.22	0.19	17.98	2,121
Chronic intermediate and immediate cause	5.89	6.96	0.14	1.83	0.17	0.04	15.03	11,552
Cardiovascular diseases	3.25	9.17	0.16	0.64	1.10	0.06	14.39	434,126
Neoplasm	4.03	5.69	0.09	2.93	0.05	0.35	13.14	246,659
Diabetes and diabetes related disorders	3.18	7.84	0.09	1.56	0.20	0.05	12.91	24,894
Acute intermediate and immediate cause	3.35	4.66	0.17	1.00	0.51	0.13	9.82	29,221
Neurological disorders	6.82	1.58	0.07	0.84	0.10	0.02	9.43	37,274
Chronic respiratory diseases	4.11	3.59	0.13	1.19	0.11	0.04	9.16	39,276
HIV	3.09	0.85	1.62	1.24	0.06	0.43	7.29	3,653
Acute respiratory infections	4.51	1.23	0.06	0.61	0.03	0.03	6.47	15,346
Poisoning and overdoses	3.45	0.84	0.21	0.72	0.05	0.02	5.29	3,024
Neonatal disorders	0.82	0.95	0.13	0.50	0.00	0.05	2.45	1,318
**Grand Total**	6.02	8.11	7.45	4.79	0.54	0.15	27.07	2,173,961

This technical appendix is reproduced and slightly modified from the most up-to-date methods related to cause-of-death modelling used in the GBD 2016, published in the *Lancet*.[1]

© 2019 Sunshine JE et al. *JAMA Network Open*.

eMethods.

Section 1: Causes of death modelling methods (CODEm)

1.1 CODEm

1.1.1 Overview of method

Cause of death ensemble modelling (CODEm) is the framework used to model most cause-specific death rates in the GBD.[2] It relies on four key components. First, all available data are identified and gathered to be used in the modelling process. Though the data may vary in quality, they all contain some signal of the true epidemiological process. Second, a diverse set of plausible models are developed to capture well-documented associations in the estimates. Using a wide variety of individual models to create an ensemble predictive model has been shown to outperform techniques using only a single model both in cause of death estimation[2] and in more general prediction applications.[3,4] Third, the out-of-sample predictive validity is assessed for all individual models, which are then ranked for use in the ensemble modelling stage. Finally, differently weighted combinations of individual models are evaluated to select the ensemble model with the highest out-of-sample predictive validity.

For some causes (see, for example, lower respiratory infections), there is evidence that the relationship between covariates and death rates might differ between children and adults. Separate models are therefore run for different age ranges when applicable. Additionally, separate models are developed for countries with extensive, complete, and representative vital registration (VR) for every cause to ensure that uncertainty can better reflect the more complete data in these locations.

1.1.2 Model pool development

Because many factors may covary with any given cause of death, a range of plausible statistical models are developed for each cause. In the CODEm framework, four families of statistical models are used: linear mixed effects regression (LMER) models of the natural log of the cause-specific death rate, LMER models of the logit of the cause fraction, spatiotemporal Gaussian process regression (STGPR) models of the natural logarithm of the cause-specific death rate, and ST-GPR models of the logit of the cause fraction (see the 2x2 table in Foreman et al).[2] For each family of models, all plausible relationships between covariates and the response variable are identified. Because all possible combinations of selected covariates are considered for each family of models, multicollinearity between covariates may produce implausible signs on coefficients or unstable coefficients. Each combination is therefore tested for statistical significance (covariate coefficients must have a coefficient with p-value $< 0{\cdot}05$) and plausibility (the coefficients must have the directions expected based on the literature). Only covariate combinations meeting these criteria are retained. This selection process is run for both cause fractions and death rates, then ST-GPR and LMER-only models are created for each set of covariates. For a detailed explanation of the covariate selection algorithm, see Foreman et al 2012.[2]

1.1.3 Testing model pool on 15% sample

The performance of all models (individual and ensemble) is evaluated using out-of-sample predictive validity tests. Thirty percent of the data are excluded from the initial model fits. These individual model fits are evaluated and ranked using half of the excluded data (15% of the total), then used to construct the ensembles based on their performance. Data are held out from the analysis based on the cause-specific missingness patterns for ages and years across locations. Out-of-sample predictive validity testing is repeated 20 times for each model, which has been shown to produce stable results.[2] These performance tests include the RMSE

© 2019 Sunshine JE et al. *JAMA Network Open*.

for the log of the cause-specific death rate, the direction of the predicted versus actual trend in the data, and the coverage of the predicted 95% UI.

1.1.4 Ensemble development and testing

The component models are weighted based on their predictive validity rank in order to determine their contribution to the ensemble estimate. The relative weights are determined both by the model ranks and by a parameter ψ, whose value determines how quickly the weights taper off as rank decreases. The distribution of ψ is described in more detail in Foreman et al 2012.[2] A set of ensemble models is then created using the weights constructed from the combinations of ranks and ψ values. These ensembles are tested using the predictive validity metrics described in Section 1.1.3 on the remaining 15% of the data, and the ensemble with the best performance in out-of-sample trend and RMSE is chosen as the final model.

1.1.5 Final estimation

Once a weighting scheme has been chosen, 1,000 draws are created for the final ensemble, with the number of draws contributed by each model proportional to its weight. The mean of the draws is used as the final estimate for the CODEm process, and a 95% uncertainty interval (UI) is created from the 0.025 and 0.975 quantiles of the draws. The validity of the UI can be checked via its coverage of the out-of- sample data; ideally, the 95% UI would capture 95% of these data. Higher coverage suggests that the UIs are too large, and lower coverage suggests overfitting.

1.1.6 Selection of causes for which CODEm is used

CODEm is used to model 205 causes, described in detail elsewhere. However, it is unsuitable for use in modelling certain causes, including those with very low death counts, those where cause-specific death record availability is inadequate, or those for which there are marked biases or variability for cause of death certification over time that cannot be fully accounted for with the current garbage code redistribution algorithms.

1.1.7 Model-specific covariates

A table of CODEm covariates used, level of the covariate, and expected direction of the covariate can be found in eTable3.

eReferences.

1. Naghavi M, Abajobir AA, Abbafati C, et al. Global, regional, and national age-sex specific mortality for 264 causes of death, 1980–2016: a systematic analysis for the Global Burden of Disease Study 2016. *The Lancet*. 2017;390(10100):1151-1210 (appendix, p33).

2. Foreman KJ, Lozano R, Lopez AD, Murray CJ. Modeling causes of death: an integrated approach using CODEm. *Popul Health Metr*. 2012; 10:1.

3. Bell RM, Koren Y. Lessons from the Netflix Prize Challenge. *SIGKDD Explor Newsl.* 2007; 9: 75–79.

4. Bell RM, Koren Y, Volinsky C. All together now: A perspective on the NETFLIX PRIZE. *CHANCE*. 2010; 23: 24–24.

© 2019 Sunshine JE et al. *JAMA Network Open*.
